# Megafaunal Communities in Rapidly Warming Fjords along the West Antarctic Peninsula: Hotspots of Abundance and Beta Diversity

**DOI:** 10.1371/journal.pone.0077917

**Published:** 2013-12-03

**Authors:** Laura J. Grange, Craig R. Smith

**Affiliations:** Department of Oceanography, University of Hawaii at Manoa, Honolulu, Hawaii, United States of America; Université du Québec à Rimouski, Canada

## Abstract

Glacio-marine fjords occur widely at high latitudes and have been extensively studied in the Arctic, where heavy meltwater inputs and sedimentation yield low benthic faunal abundance and biodiversity in inner-middle fjords. Fjord benthic ecosystems remain poorly studied in the subpolar Antarctic, including those in extensive fjords along the West Antarctic Peninsula (WAP). Here we test ecosystem predictions from Arctic fjords on three subpolar, glacio-marine fjords along the WAP. With seafloor photographic surveys we evaluate benthic megafaunal abundance, community structure, and species diversity, as well as the abundance of demersal nekton and macroalgal detritus, in soft-sediment basins of Andvord, Flandres and Barilari Bays at depths of 436–725 m. We then contrast these fjord sites with three open shelf stations of similar depths. Contrary to Arctic predictions, WAP fjord basins exhibited 3 to 38-fold greater benthic megafaunal abundance than the open shelf, and local species diversity and trophic complexity remained high from outer to inner fjord basins. Furthermore, WAP fjords contained distinct species composition, substantially contributing to beta and gamma diversity at 400–700 m depths along the WAP. The abundance of demersal nekton and macroalgal detritus was also substantially higher in WAP fjords compared to the open shelf. We conclude that WAP fjords are important hotspots of benthic abundance and biodiversity as a consequence of weak meltwater influences, low sedimentation disturbance, and high, varied food inputs. We postulate that WAP fjords differ markedly from their Arctic counterparts because they are in earlier stages of climate warming, and that rapid warming along the WAP will increase meltwater and sediment inputs, deleteriously impacting these biodiversity hotspots. Because WAP fjords also provide important habitat and foraging areas for Antarctic krill and baleen whales, there is an urgent need to develop better understanding of the structure, dynamics and climate-sensitivity of WAP subpolar fjord ecosystems.

## Introduction

Fjords are deep estuaries carved by glaciers and typically contain one or more sediment-floored basins separated by sills [1], [2]. Fjords with tidewater glaciers (glacio-marine fjords) are widespread at temperate to polar latitudes and form important boundary zones between the cryosphere and the ocean [3]. At high-latitudes, such as in the polar-tundra (or subpolar) climate conditions of arctic Canada, coastal Greenland, Svalbard and the West Antarctic Peninsula (WAP), fjord ecosystems serve as major conduits for glacial ice to the sea and are thus highly sensitive to cryosphere-ocean interactions and to climate warming [4], [5], [6]. Because of their distinct geomorphology, circulation processes, and terrigenous inputs (including glacial ice, meltwater and sediments), glacio-marine fjords can exhibit substantially different ecosystem forcing than adjacent continental shelves. Fjord ecosystem studies, conducted mostly outside Antarctica, indicate that fjords may contain intense ecological disturbance gradients, unusual food-web structure, genetically isolated populations, and refugia for cold-adapted species [5], [6], [7], [8], [9], [10], [11], [12], [13], [14], [15], [16].

**Figure 1:**
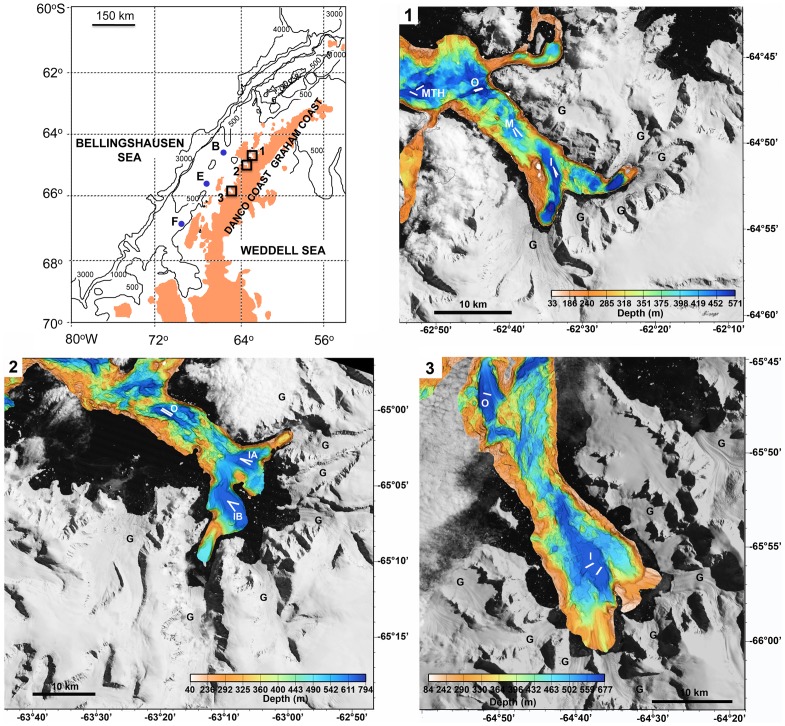
Distribution of sampling sites in subpolar fjords and open shelf stations along the WAP. Blue dots are shelf stations B, E and F, at depths of ∼600 m; boxes indicate the subpolar WAP fjords (**1**) Andvord, (**2**) Flandres and (**3**) Barilari Bays. Panels 1–3: multibeam bathymetry superimposed on satellite imagery of the three WAP fjords. White lines indicate phototransect positions: I = inner basin (IA = inner basin A & IB = inner basin B); M = middle basin; O = outer basin; and MTH = fjord mouth. ‘G’ indicates the location of a tidewater glacier. Note that each fjord has multiple tidewater glaciers 10–15 km long carrying ice from the Peninsula ice cap (previously described by Cook et al. [31]). Data available from the U.S. Geological Survey. Satellite images are public domain USGS Products.

The WAP, including the Danco/Graham Coast (Figure 1), harbors the most extensive system of glacio-marine fjords on the Antarctic continent. While the glacio-marine settings of the subpolar fjords along the Danco/Graham Coast are relatively well studied from a geological perspective [3], [17], [18], [19], [20], ecosystem structure and function in these subpolar fjords remain very poorly evaluated despite their potential to provide climate-sensitive habitats along the Antarctic margin for keystone species such as krill and their predators [21], [22], [23], [24].

In the Arctic (e.g., Svalbard, Baffin Island, Greenland), ecosystem structure and function of subpolar glacio-marine fjords have been extensively evaluated, revealing heavy influence from meltwater processes and turbidity plumes [3], [5], [6], [7], [8], [10], [13], [25], [26], [27]. The inner-middle portions of these Arctic fjords (e.g., within 5–10 km of tidewater glaciers) typically sustain high water-column turbidity and sedimentation rates (e.g., 2–25 cm y^−1^) limiting primary production and yielding substantial burial disturbance of fjord macro- and megabenthos [4], [5], [6], [8], [10], [13], [28], [29] (Table S1). In the inner-middle basins, low-diversity assemblages of small bodied, motile deposit feeders adapted to chronic burial disturbance characterize macro- and megabenthic communities. Biomass, species richness, and trophic complexity of macrofauna and megafauna have been shown to increase towards the fjord mouth, where sedimentation rates decline and labile organic-carbon content in sediments rises [4], [5], [10], [13], [25], [29], [30]. Thus, strong meltwater processes and terrigenous sedimentation in Arctic subpolar fjords cause inner-middle basins to be diversity and productivity “coldspots” [8], [10], [13]. Similar down-fjord gradients of burial disturbance and macrobenthic community structure have been documented for Eczurra Inlet of Admiralty Bay [14], [15], [16], a temperate, glaciated fjord on King George Island, north of the Antarctic Peninsula [20]. Time series and latitudinal studies of Arctic fjords ([4], [5] respectively), suggest that climate warming and glacial retreat onto land will reduce glacial disturbance of many Arctic fjords, yielding increases in fjord productivity, macro- and megabenthic standing crop, and seafloor biodiversity.

The WAP, including its subpolar fjords, is warming as rapidly as anywhere on earth, with mean winter air temperatures rising by 6°C, sea-surface temperatures increasing by >1°C, and 87% of tidewater glaciers having retreated over the past 50 y [21], [31], [32]. Can we apply the benthic ecosystem models of Arctic subpolar fjords [4], [5] to WAP subpolar fjords to predict the effects of climate warming? If the Arctic models apply, we expect the following hypotheses concerning benthic ecosystem structure to apply in subpolar WAP fjords:

*Benthic communities in the inner-middle basins of WAP subpolar fjords (i.e., within ∼5–10 km of tidewater glaciers) are characterized by low (a) faunal abundance, (b) species richness, (c) species diversity, and (d) trophic complexity, as well as (e) dominance by small-bodied mobile deposit feeders, compared to similar depths on the open WAP shelf.*
*(a) Faunal abundance, (b) species richness, (c) species diversity, and (d) trophic complexity increase down fjord from the inner fjord basins to fjord mouths, as well as with distance from glacial termini.*

Here we use seafloor photographic surveys to test these predictions for epibenthic megafaunal communities in three WAP subpolar fjords that are bordered by numerous tidewater outlet glaciers, comparing community structure between fjords and the open continental shelf. In contrast to predictions from Arctic fjord models, we find that communities in the inner-middle basins of these WAP fjords are hotspots of megabenthic abundance and harbor trophically complex, species-rich assemblages distinct from the open shelf. We discuss the reasons for these benthic community differences between Arctic and Antarctic subpolar fjords, and suggest that climate warming may have dramatic negative impacts on benthic abundance and biodiversity in at least some Antarctic subpolar fjords, altering regional patterns of benthic beta and gamma diversity.

### Study Sites and Methods

#### (a) Study Sites

We studied benthic megafaunal and demersal nekton communities in the sediment-floored basins in three subpolar fjords along the Danco/Graham Coast of the WAP: Andvord, Flandres and Barilari Bays. We then compared these fjord communities to assemblages at similar latitudes and depths along the open WAP shelf (Figure 1). The three fjords are similarly sized with multiple basins at depths of ∼500–700 m, the typical depth of the open WAP shelf. Tidewater glacial termini, including multiple outlet glaciers from the Antarctic Peninsula ice sheet, occupy at least 30–40% of the shorelines of all three fjords [3]; thus, they all sustain large ice inputs from tidewater glaciers [3], [20]. Andvord and Flandres Bays have been free of large ice shelves for >3000 y [33], and nearly all of Barilari Bay has been open for >100 y [34]; thus, all three fjords have had at least a century for marine ecosystem development.

The Danco/Graham Coast experiences a cold, dry climate characterized as subpolar, with mean summer temperatures of ∼0°C [18] and mean annual temperatures within the range of −3 to +4°C [3], [33]. As a consequence of this climate, these WAP fjords experience limited glacial melt, with glacier equilibrium lines occurring near sea level [3], [18], [20], [33].

#### (b)Seafloor surveys

Seafloor photosurveys in fjord basins were conducted over a two-month period on the RVIB N. B. Palmer in 2010 (cruise NBP10-01) at ten stations at depths of 436–725 m in Andvord, Flandres and Barilari Bays (Table S2). Seafloor images at the three open shelf stations (Stations B, E and F; Figure 1) were taken at comparable depths (573–678 m) over a thirteen-month period from the ASRV L. M. Gould and RVIB N.B. Palmer during the FOODBANCS2 Project [35] (Figure 1, Table S2). All fjord and shelf stations consisted of flat muddy sediments, although the frequency of dropstones was substantially higher in fjords.

Photographic surveys were conducted using a vertically downward looking “Yoyo Camera” system developed for the FOODBANCS2 Project. This Yoyo Camera system consisted of a tubular steel frame supporting an Ocean Imaging Systems DSC 10000 digital still camera in titanium housing (10.2 megapixel, 20-mm, Nikon D-80 Camera), with an Ocean Imaging Systems 3831 Strobe (200 W-S) located 1-m from the camera at an angle of 26° from vertical, and a Model 494 Bottom Contact Switch (Figure S1). Camera settings were: F-8, Focus 1.9 m, ASA-400. The Yoyo Camera system was deployed on a coaxial cable and included a transmissometer and audible contact alarm providing real-time information on turbidity levels around the camera and the moment of bottom-switch contact. The alarm and transmissometer allowed us to collect bottom images at a high rate without dragging the camera, even in the rough seas characteristic of the open WAP shelf. Images were digitally color corrected (blue bias removed) using Adobe ImageReady software, based on *in situ* photographs of a color chart. The camera and strobe were actuated at 2.5 m above the seafloor by bottom contact switch, imaging ∼3 m^2^ of seafloor. Parallel laser beams (10-cm separation) established scale in images. Megafauna and sediment structures down to 1–2 cm in largest dimension were resolvable.

At each station, with the exception of the outer basin of Barilari Bay, two randomized photosurvey transects were conducted; only one phototransect was possible in outer Barilari Bay due to shiptime constraints. Within fjords, phototransects started at randomly selected points within a fjord basin, with photosurveys then conducted along the long axis of the basin. For open shelf stations, each transect began within ∼100 m of the central station location (Table S2) and proceeded along a line within ∼20° of a random heading (heavy winds or sea ice required up to 20° modification of line directions in some cases). During tows, the Yoyo Camera system was lowered to the seafloor with the ship holding station; after contact the system was towed at ∼1 knot, raising and lowering the system approximately 2 m between firings. Time intervals between photographs were ∼15 s, yielding a spacing of 5–10 m between consecutive photographs. Phototransects were terminated after transiting a distance of >1 km and obtaining >100 bottom images.

#### (c) Epibenthic megafaunal analysis

From each transect, all seabed photographs were viewed and a comprehensive species atlas was created of the identifiable epibenthic megafauna at each site. We counted epibenthic megafauna only on soft sediments to allow within-habitat comparisons across fjord basins and the open shelf. Dropstones with hard-substrate epifauna were extremely rare on the open shelf and covered only a few percent of the seafloor in the fjords; this hard substrate fauna will be the focus of a future study. We also tabulated the occurrence of drift macroalgae on the seafloor, and the abundance of demersal nekton species visible in photographs. Megafaunal samples collected by Blake trawl were used to aid identification of organisms in seafloor images. All collections were made in international waters, under the auspices of, and with permission from, the United States Antarctic Program (USAP). Marine finfish and invertebrates only were collected by Blake trawl, towed for 0.5 hr on the seafloor. No endangered or protected species were collected in this study. Trawl-collected marine fish and invertebrates were humanely sacrificed by rapid freezing, or by rapid warming to room temperature (which anesthetizes Antarctic marine benthos adapted to living at −1.0°C). Field collections of fish and invertebrates within the USAP do not require IACUC approval.

Megafauna in photographs were identified to the lowest possible taxon, typically to species. A number of putative “species” appeared morphologically distinct in seafloor photographs but could not be confidently related to a described species; these “species” were assigned a unique species number (e.g., Cerianthid sp. 1). Some individuals could only be resolved to coarse taxonomic levels (i.e., ophiuroid or polychaete); these were not included in analyses of community structure and diversity.

Fifty photographs of suitable quality (i.e., camera vertical and view clear of suspended sediments) were drawn at random, using the RANDBETWEEN function in Microsoft Excel, for analysis from each transect. ImageJ ® software was used to scale individual images using the laser marks on the seafloor, and then to count all species of epibenthic megafauna and demersal nekton within a 1.8 m^2^ area in the image center.

#### (d) Distance to glacial termini and down-fjord measurements

The distance from phototransect midpoints to the nearest tidewater glacial terminus [31] was measured in Google Earth. Because 30–40% of the fjord shorelines are occupied by glacial termini (Figure 1), the nearest glacial terminus was not necessarily located at the head of the fjord. Therefore, photosurveys were also ordered along a down-fjord gradient of inner basin, middle basin, outer basin and fjord mouth (where possible) to explore down-fjord effects.

#### (e) Statistical analyses

There are two natural scales of sampling in our phototransect data; the scale of individual photographs (1.8 m^2^) randomized within phototransects, and the scale of phototransects (a linear scale of ∼1 km and a total area of ∼90 m^2^) randomized within stations. Low faunal numbers, including zeros, in many individual photographs on the open shelf made community analyses problematic at the individual photograph (1.8 m^2^) scale. To maintain consistency across analyses, we used phototransects as our sampling unit. Because positions of individual transects were randomized, we treated phototransects within a fjord basin or open shelf station as spatially independent. Transects were then grouped at the local scale (i.e., within fjord basin or open shelf station), fjord scale (within whole fjord or open shelf station), and regional scale (pooled fjords versus pooled open shelf stations), to explore fjord contributions to alpha, beta and gamma diversity, and to explore patterns of community structure among habitats.

Minitab 15.0 was used to test statistical differences in megafaunal abundance and biodiversity metrics (Shannon's H′, Hurlbert rarefaction Es(100), mean number of species per 90 m^2^ phototransect, Pielou's Evenness J′, and Chao 1 species richness) between whole fjords and open shelf stations B, E and F (plus the open shelf as a whole). These data, even after log transformations, violated assumptions of normality. Therefore, the non-parametric Kruskal-Wallis test and multiple Mann Whitney comparisons were used, and P values adjusted with a Bonferroni correction.

We explored patterns of megafaunal community structure (1) down-fjord, by grouping samples by fjord basin (inner, middle, outer and mouth), and (2) among fjord and shelf stations, by grouping samples by whole fjord (Andvord, Flandres and Barilari Bays) and open shelf stations (Stations B, E and F). Similarity between communities was compared using Primer 6.0 software [36] across all phototransects based on non-metric multi-dimensional scaling (nMDS), using 4^th^ root transformed data (to allow contributions from common and rare species) and Bray-Curtis similarity [37], [38]. Because the nMDS plot yielded a stress value of 0.15, we confirmed the nMDS patterns using Bray Curtis Group Averaging cluster analyses, as recommended by Clarke & Warwick [38]. Analysis of similarities (ANOSIM) was used to determine the statistical significance and validity of differences in community structure among fjord and open shelf station groups [37]. SIMPER (Similarity percentage; [37]) analysis was used to explore which taxa were primarily responsible for observed differences between whole fjords and shelf stations. For functional group analyses, species were assigned to trophic categories (suspension feeder, deposit feeder, carnivore/predator and scavenger/omnivore) and mobility groups (sessile and mobile) based on seafloor photographs, ROV observations, examination of gut material, and the scientific literature.

To explore patterns of diversity at the local scale, we used the following biodiversity metrics: (1) Shannon's H′ and Hurlbert rarefaction Es(100) for total species diversity (evenness+species richness), (2) species density, i.e., mean number of species per 90 m^2^ phototransect (species richness), (3) Pielou's Evenness J′ (evenness) and (4) Chao 1 species richness (estimated total species richness). For comparisons of observed species richness across local, fjord and regional scales, we used Ugland species accumulation curves to determine whether species inventories at a particular scale were complete (i.e., approached asymptotic values) or were continuing to accumulate [39]. Since species were accumulating at all scales (see figures in electronic supporting information), we then followed the recommendations of Magurran [40] and used the species richness estimators Chao 1, Bootstrap and Jackknife 2 to estimate total species richness (i.e., the species richness expected from complete sampling of the assemblage) at the various scales. The contributions of fjords to beta diversity were assessed by determining the number of species unique to fjords, and by estimating total species richness for pooled fjord transects, pooled shelf transects, and for all fjord and shelf transects combined using Chao 1, Bootstrap and Jackknife 2 species richness estimators, as in [41]. Diversity analyses were conducted in Primer 6.0 software [36].

## Results

**Figure 2:**
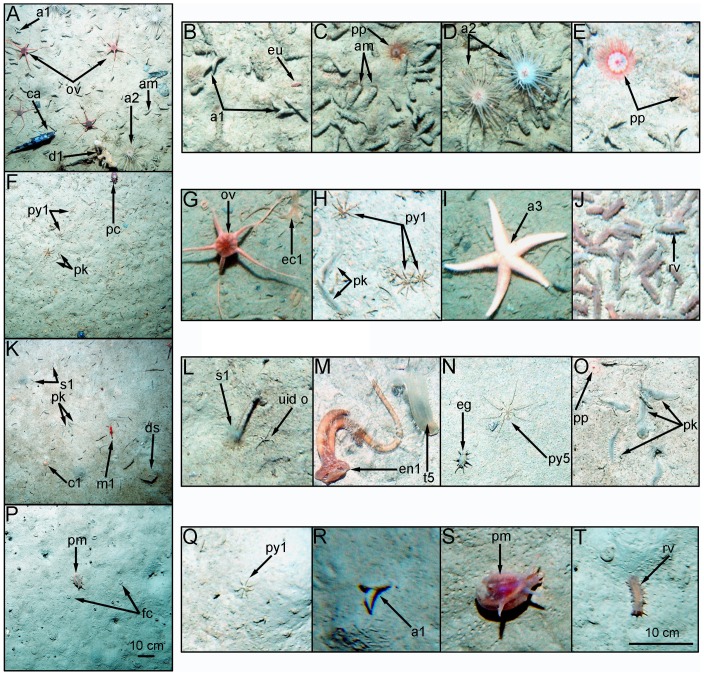
Representative images of the seafloor and dominant epibenthic megafauna in fjords and on the open shelf. (**A**) Typical view of Andvord Bay middle basin and (**B–E**) dominant megafauna of Andvord Bay. (**F**) Typical view of Flandres Bay inner basin A and (**G–J**) dominant megafauna of Flandres Bay. (**K**) Typical view of Barilari Bay outer basin and (**L–O**) dominant megafauna of Barilari Bay. (**P**) Typical view of open shelf Stn F (summer 2009) and (**Q–T**) dominant megafauna of the open shelf. Faunal identifications are as follows: a1 = Ampeliscid amphipod sp. 1; a2 = Anemone sp. 2; a3 = Asteroid sp. 3 (*Diplasterias brucei*?); am = *Amythas membranifera*; c1 = Cup coral sp. 1; ca = *Chaenocephalus aceratus*; d1 = Demospongiae sp. 1; ds = Dropstone; ec1 = Bonellid echiuran sp. 1 proboscis; eg = *Elpidia glacialis*; en1 = Enteropneust sp. 1; eu = Eusirid sp.; fc = Fecal coils of *Protelpidia murrayi*; m1 = Mysid sp. 1; ov = *Ophionotus victoriae*; pc = *Pareledone charcoti*; pk = *Prionosyllis kerguelensis*; pm = *Protelpidia murrayi*; pp = *Ptychogastria polaris*; py1 = Pycnogonid sp. 1; py5 = Pycnogonid sp. 5; rv = *Rhipidothuria racovitzai*; s1 = Sabellid sp. 1; t5 = Tunicate sp 5; and uid o = UID ophiuroid. Note that the panels (**A, F, K** and **P**) share the scale indicated in (**P**), and panels (**B–E, G–J, L–O**, and **Q–T**) share the scale indicated in (**T**).

We counted a total of 42,202 benthic megafaunal individuals from 12 phyla and 116 putative species; 39,381 individuals from 91 nominal taxa in fjords, and 2821 individuals from 77 taxa from the WAP open shelf stations (see Figure 2 for representative taxa). In addition, we counted 1897 demersal nekton individuals from 3 phyla and 6 nominal taxa; 1533 individuals in fjords and 364 from shelf stations (see Figure 2 for some examples of representative taxa).

### (a) Patterns of megafaunal abundance

**Figure 3:**
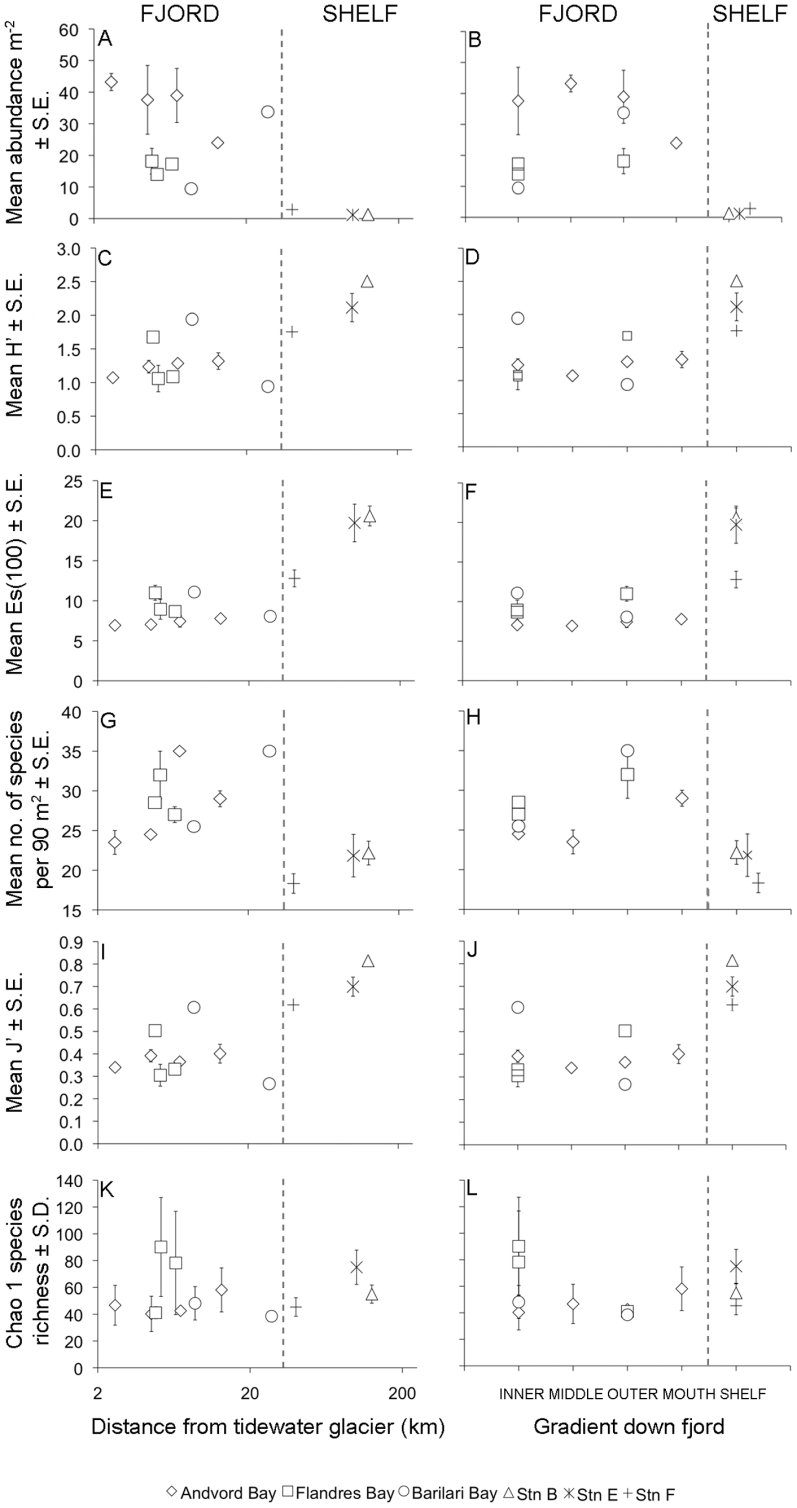
Epibenthic megafaunal abundance and diversity at the local scale. Data are plotted as a function of distance to the nearest tidewater glacier, and position in basins down fjord. (**A–B**) Epibenthic megafaunal abundance (m^−2^), (**C–D**) Shannon's H′, (**E–F**) Hurlbert rarefaction Es(100), (**G–H**) Number of species per 90 m^2^ phototransect (**I–J**) Pielou's Evenness J′, and (**K–L**) Chao 1 species richness for fjord basins and open shelf stations.

Mean megafaunal abundance at fjord stations was 3 to 38-fold greater than mean megafaunal abundance at open shelf stations (Figure 3A & B), with all fjord-shelf differences highly statistically significant (P≤0.001) (Table 1). Overall, average megafaunal community abundance in fjords was more than 15-fold higher than the open shelf average. In Andvord and Flandres Bays, megafaunal community abundances remained very high in close proximity (<5 km) to the termini of tidewater glaciers (Figure 3A), and showed no evident trend of declining abundance from outer to inner basins (Figure 3B). In Barilari Bay, megafaunal abundance declined from the outer to inner basin, and with proximity to tidewater glaciers; nonetheless, abundance in inner Barilari Bay still remained 3 to 8-fold higher than at any open shelf station.

**Table 1:** Differences in epibenthic megafaunal abundance, species diversity, species density, evenness and estimated total species richness between fjords and open shelf stations.

	Abundance		Species diversity				Species density		Evenness		Estimated total species richness	
		Mean (m^−2^)		Shannon Diversity (H'loge)		Hurlbert Rarefaction Diversity (Es(100))		Mean number of species per 90 m^2^ phototransect		Pielou's evenness (J′)		Chao 1
**Andvord Bay**												
Fjord vs open shelf stations B, E & F	+	***	−	***	−	***	+	*	−	***	+	**
Fjord vs Stn B	+	**	−	**	−	**	+	0.0312	−	**	+	N.S.
Fjord vs Stn E	+	**	−	**	−	**	+	N.S.	−	**	−	0.0332
Fjord vs Stn F	+	**	−	**	−	**	+	**	−	**	+	0.0332
**Flandres Bay**												
Fjord vs open shelf stations B, E & F	+	***	−	**	−	**	+	**	−	***	+	**
Fjord vs Stn B	+	**	−	**	−	**	+	**	−	**	+	0.0202
Fjord vs Stn E	+	**	−	0.0131	−	**	+	0.0442	−	**	+	N.S.
Fjord vs Stn F	+	**	−	0.0453	−	0.0202	+	**	−	**	+	0.0306
**Barilari Bay**												
Fjord vs open shelf stations B, E & F	+	**	−	*	−	*	+	*	−	**	−	*
Fjord vs Stn B	+	0.0282	−	0.0282	−	0.0282	+	0.0269	−	0.0282	+	N.S.
Fjord vs Stn E	+	0.0282	−	N.S.	−	0.0282	+	N.S.	−	N.S.	−	N.S.
Fjord vs Stn F	+	0.0282	−	N.S.	−	N.S.	+	0.0201	−	N.S.	+	N.S.

“+”; lower values in the fjords by a “−”. Differences between fjords and the open shelf as a whole (Stations B, E & F) were tested using the Kruskal-Wallis test. Pairwise comparisons between individual fjords and individual open shelf stations were addressed using multiple Mann-Whitney U tests. ****P<0.0001, ***P<0.001, **P<0.01, *P<0.05 and N.S. (Non-significant) P>0.05. Numbers indicate probability values that were <0.05 but exceeded the alpha level calculated with a Bonferroni correction. Higher values in the fjords are indicated by a

### (b) Patterns of megafaunal community structure

#### Dominant species

The dominant megafaunal species in fjords were generally much more abundant, and frequently came from different species, than those on the open shelf (Figure 2 & Table 2). There were also substantial between-fjord differences in dominant species. Andvord Bay was dominated throughout by the tube-building ampharetid polychaete *Amythas membranifera* and an ampeliscid amphipod which together accounted for >74% of megafaunal abundance. Individuals of *A. membranifera* were generally much larger in body size than ampharetids trawled on the open shelf, and many *A. membranifera* from Andvord Bay were gravid, with bodies distended with eggs or sperm (Figure S2). Flandres Bay was heavily dominated by Pycnogonid sp. 1 and the ophiuroid *Ophionotus victoriae* in one inner basin (Figure 2G & H), with the polychaete *Prionosyllis kerguelensis* (Figure 2H) replacing *O. victoriae* as co-dominant in the other Flandres Bay basins. Flandres Bay also exhibited small-scale spatial heterogeneity in dominant species; the holothurian *Rhipidothuria racovitzai* reached an extraordinary density of 623 individuals m^−2^ in one photograph in outer Flandres Bay (Figure 2J). The inner basin of Barilari Bay exhibited substantial dominance by the polychaete Sabellid sp. 1 (Figure 2L), as well as by a large pycnogonid and the holothurian *Elpidia glacialis* (Figure 2N).

**Table 2:** Dominant epibenthic megafaunal species by percentage of total abundance in WAP fjord basins.

FJORD BASINS							
**AI**	**Mean**	**S.E.**	**%**	**FIA**	**Mean**	**S.E.**	**%**
*Amythas membranifera*	20.2	6.13	53.8	Pycnogonid sp. 1	9.7	1.62	69.1
Ampeliscid amphipod sp. 1	11.4	4.89	30.3	*Prionosyllis kerguelensis*	3.0	0.86	21.7
Anemone sp. 2	3.5	0.50	9.2	Anemone sp. 5	0.2	0.09	1.5
*Ptychogastria polaris*	1.0	0.02	2.6	*Ptychogastria polaris*	0.2	0.08	1.2
	0.3	0.32	0.8	*Pareledone charcoti*	0.1	0.02	0.8
**AM**	**Mean**	**S.E.**	**%**	**FIB**	**Mean**	**S.E.**	**%**
*Amythas membranifera*	28.5	2.16	66.1	Pycnogonid sp. 1	12.2	0.95	70.3
Ampeliscid amphipod sp. 1	9.6	0.67	22.2	*Ophionotus victoriae*	3.1	0.55	18.0
Anemone sp. 2	2.3	0.14	5.4	Asteroid sp. 3 (*Diplasterias brucei?*)	0.5	0.00	2.6
*Ptychogastria polaris*	0.9	0.29	2.1	Asteroid sp. 2 (small & white)	0.4	0.06	2.6
*Ophionotus victoriae*	0.6	0.31	1.5	Demospongiae sp. 1	0.3	0.04	1.8
**AO**	**Mean**	**S.E.**	**%**	**FO**	**Mean**	**S.E.**	**%**
Ampeliscid amphipod sp. 1	21.4	3.54	55.0	*Prionosyllis kerguelensis*	6.6	0.03	36.4
*Amythas membranifera*	10.1	4.55	26.0	Pycnogonid sp. 1	4.5	0.54	25.0
*Ptychogastria polaris*	4.2	0.63	10.8	*Rhipidothuria racovitzai*	3.5	3.46	19.2
Anemone sp. 2	1.7	0.12	4.3	Anemone sp. 4	0.9	0.06	5.0
Hydroid sp. 1	0.2	0.02	0.6	Anemone sp. 5	0.8	0.03	4.2
**AMTH**	**Mean**	**S.E.**	**%**	**BI**	**Mean**	**S.E.**	**%**
Ampeliscid amphipod sp. 1	12.8	3.19	53.4	Sabellid sp. 1	2.3	0.49	23.8
*Amythas membranifera*	5.0	3.34	20.8	Pycnogonid sp. 5 (large & spindly)	2.2	0.27	22.9
*Ptychogastria polaris*	2.9	0.18	12.3	*Elpidia glacialis*	1.5	0.59	16.1
Anemone sp. 2	2.1	0.02	8.6	Pycnogonid sp. 1	1.5	0.31	16.1
Terebellid sp. 1	0.1	0.02	0.6	Tunicate sp. 5	1.1	0.08	11.2
				**BO**	**Mean**	**S.E.**	**%**
				Sabellid sp. 1	25.8	0.00	76.5
				*Prionosyllis kerguelensis*	4.7	0.00	13.8
				Pycnogonid sp. 1	0.9	0.00	2.7
				Irregular urchin sp. 1	0.5	0.00	1.4
				Cup coral sp. 1	0.3	0.00	1.0

^−2^ ± 1 SE using phototransects as replicates. Basins: AI = Andvord Bay inner; AM = Andvord Bay middle; AO = Andvord Bay outer; AMTH = Andvord Bay mouth; FIA = Flandres Bay inner A; FIB = Flandres Bay inner B; FO = Flandres Bay outer; BI = Barilari Bay inner; and BO = Barilari Bay outer. Species listed are the top five by percentage abundance in WAP fjord basins. Data are mean abundances m

Open shelf stations shared four dominant taxa with the fjords (i.e., Pycnogonid sp. 1, Ampeliscid amphipod sp. 1, *E. glacialis*, and *R. racowtizai*; Figure 2Q, R & T and Table 3), but the abundance of dominant species was invariably much lower on the shelf.

**Table 3:** Dominant epibenthic megafaunal species by percentage of total abundance at WAP open shelf stations.

OPEN SHELF STATIONS							
**B**	**Mean**	**S.E.**	**%**	**E**	**Mean**	**S.E.**	**%**
Pycnogonid sp. 1	0.3	0.05	21.3	Ampeliscid amphipod sp. 1	0.4	0.06	31.7
Ophiuroid sp. 5 (Small, blue central disc)	0.2	0.04	13.1	*Protelpidia murrayi*	0.2	0.02	16.7
Tunicate sp. 8 (*Synoicum* sp?)	0.1	0.06	10.3	*Elpidia glacialis*	0.1	0.06	11.9
Cerianthid sp. 1	0.1	0.03	8.4	Pycnogonid sp. 1 complex	0.05	0.02	4.2
Tunicate sp. 4	0.1	0.02	7.6	Tunicate sp. 8 (*Synoicum* sp?)	0.04	0.03	3.5
**F**	**Mean**	**S.E.**	**%**				
*Rhipidothuria racovitzai*	1.0	0.18	36.8				
Ampeliscid amphipod sp. 1	0.6	0.05	21.1				
*Protelpidia murrayi*	0.6	0.04	19.6				
Munnopsid sp. 2	0.1	0.02	4.3				
*Peniagone vignoni*	0.1	0.01	3.1				

^−2^ ± 1 SE using individual phototransects as replicates. Species listed are the top five by percentage abundance at open shelf stations. Data are mean abundances m

Some fjord basins had a remarkable abundance of species rarely observed on the open shelf, including the benthic trachymedusa, *Ptychogastria polaris* (Figure 2E), which attained densities of 0.9–4.2 m^−2^ in Andvord Bay basins, but was not recorded on the open shelf. A medusa similar in appearance to the epibenthic individuals was common in the Andvord Bay demersal nekton (Table S3), suggesting active recruitment of this medusa to the benthos. The large acorn worm, Enteropneust sp. 1 (Figure 2M), was also 36-fold more abundant in inner Barilari Bay (0.07 m^−2^) than on the open shelf.

#### nMDS Analyses

**Figure 4:**
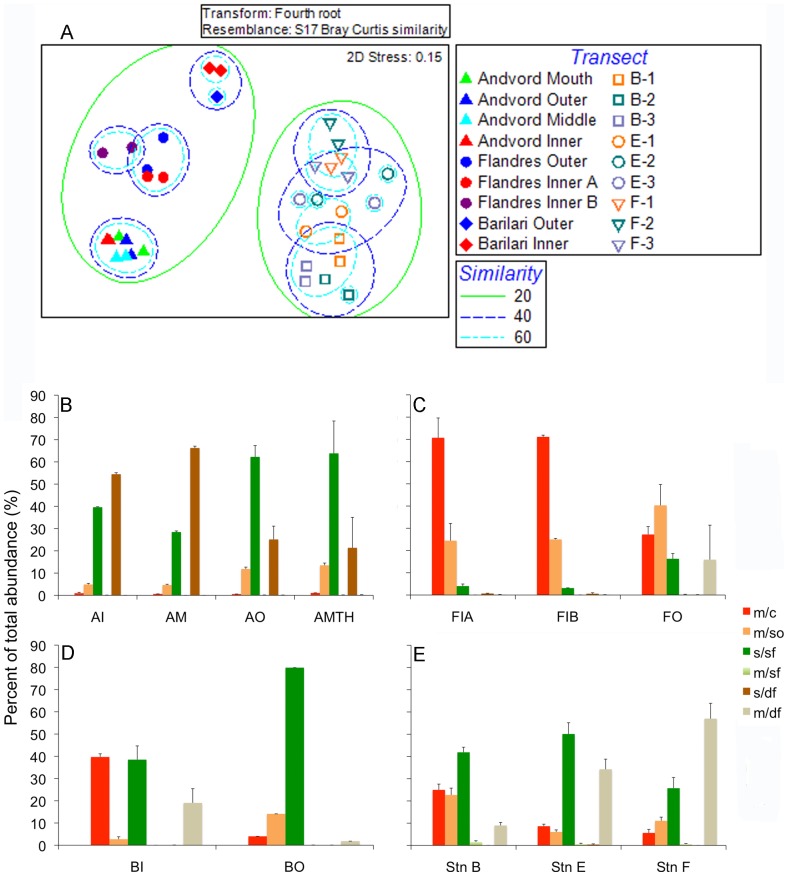
Patterns of epibenthic megafaunal community structure. (**A**) Non-metric multidimensional scaling (nMDS) plot of the Bray-Curtis similarity of WAP fjord and open shelf phototransects based on species composition. Phototransects from open shelf stations have been grouped by station and sampling cruise/time of collection and are indicated as follows: B1 = Station B summer 2008; B2 = Station B winter 2008; B3 = Station B summer 2009; E1 = Station E summer 2008; E2 = Station E winter 2008; E3 = Station E summer 2009; F1 = Station F summer 2008; F2 = Station F winter 2008; and F3 = Station F summer 2009. Also note in panels B–E, fjord basins are indicated as follows: AI = Andvord Bay inner; AM = Andvord Bay middle; AO = Andvord Bay outer; AMTH = Andvord Bay mouth; FIA = Flandres Bay inner A; FIB = Flandres Bay inner B; FO = Flandres Bay outer; BI = Barilari Bay inner; and BO = Barilari Bay outer. (**B–E**) Distributions of megafauna among functional groups (% of total abundance) in (**B**) Andvord, (**C**) Flandres and (**D**) Barilari Bays, and at (**E**) open shelf stations B, E and F. m/c = mobile carnivores/predators; m/so = mobile scavengers/omnivores; s/sf = sessile suspension feeders; m/sf = mobile suspension feeders; s/df = sessile deposit feeders; and m/df = mobile deposit feeders.

Non-metric multidimensional scaling (nMDS) and cluster analyses revealed strong community differences between fjords, and between fjord and shelf stations. Transects from individual fjords grouped tightly with two-dimensional nMDS, with substantial separation among fjords, and between fjord and open shelf stations (Figure 4A). In contrast, shelf stations showed broader similarity, with no shelf station exhibiting distinct separation. Cluster analysis supported the nMDS patterns (Figure S3). Thus, each fjord appeared to have an assemblage broadly distributed within the fjord, but distinctly different from assemblages in other fjords 30–160 km away, and substantially different from assemblages at similar depths on the open shelf as little as 70 km away. In contrast, the open shelf communities exhibited substantial similarities over much broader spatial scales, i.e., over the scales of 150–300 km that separate Stations B, E and F (Figure 4A).

ANOSIM analysis indicated community differences highly consistent with our interpretations of nMDS and cluster analyses (Table S4). There were statistically significant community differences when transects were grouped by fjords and shelf stations (Global *R* statistical = 0.964, P<0.001). Pairwise tests indicated significant differences between fjords (ANOSIM P<0.012 in all fjord versus fjord comparisons) and between each fjord and the shelf stations (ANOSIM P<0.012 in fjord versus shelf station comparisons). SIMPER analyses revealed that the characteristic species of each fjord community were largely different (Table S5, S6, S7, S8), with only *P. polaris* (in Andvord and Flandres Bays) and Pycnogonid sp. 1 (in Flandres and Barilari Bays) falling within the five most typical species in more than one fjord. SIMPER analyses also indicated that *A. membranifera*, *P. polaris*, and Ampeliscid amphipod sp. 1 were most important in distinguishing Andvord assemblages from open shelf stations; Pycnogonid sp. 1 and *Prionosyllis kerguelensis* were most important in distinguishing Flandres Bay communities from the open shelf; and a number of taxa, including Sabellid sp. 1 and *E. glacialis*, contributed to dissimilarities between Barilari Bay and open shelf communities. In summary, there were relatively few characteristic species shared across fjords, and each fjord had largely different species distinguishing its assemblage from open shelf communities (Table S9).

#### Functional group analyses

Functional-group structure differed between fjords, and between fjords and the open shelf stations (Figure 4B–E). Sessile deposit feeders and sessile suspension feeders dominated the Andvord Bay megafauna, while mobile predators, mobile scavengers/omnivores and sessile suspension feeders dominated Flandres and Barilari Bays. In contrast, open shelf stations generally had a high abundance of mobile deposit feeders, as well as sessile suspension feeders.

The high proportion of sessile deposit feeders in Andvord Bay resulted from the abundance of the ampharetid polychaete *A. membranifera*. Large numbers of ampeliscid amphipods, anemones, sponges and hydroids accounted for the high proportion of sessile suspension feeders in Andvord Bay, whereas sabellid polychaetes and a tunicate contributed most to suspension feeder proportions in Barilari Bay, particularly in the outer basin. Similar megafaunal taxa, including ampeliscid amphipods, contributed to the high proportion of sessile suspension feeders on the open shelf at stations E and F. The abundant mobile carnivores in Flandres Bay, particularly in the inner basins (∼70%), were dominated by Pycnogonid species 1. Several fish taxa, two species of polynoid scale worm, the octopus *Pareledone charcoti* and the gastropod *Harpovoluta charcoti*, also contributed to the mobile predator abundance in Flandres Bay. Mobile deposit feeders on the open shelf, particularly at stations E and F, were dominated by the surface feeding holothurians *Protelpidia murrayi*, *E. glacialis*, and *R. racovitzai*.

It is important to note that the mobile deposit feeders constituted a minor component of the fjord megabenthos, accounting for <20% (usually much less) of megafaunal abundance in all fjords. This was especially true in Andvord and Flandres Bays, where mobile deposit feeders were absent from inner basins, and showed no increasing trend with proximity to glacial termini. Incidentally, the mobile deposit feeders counted in the outer basin of Flandres Bay were mainly the 623 individuals of the holothurian *R. racovitzai* counted in a single frame (Figure 2J). Finally, trophic complexity was not markedly reduced in fjords relative to the open shelf, with all fjord basins harboring sessile suspension feeders and mobile scavengers/omnivores, and in many cases, mobile carnivores (Figure 4B–D). In addition, there was no decrease in trophic complexity with proximity to glacial termini or distance up fjord, with all fjord basins harboring at least four functional groups.

### (a) Patterns of megafaunal diversity

Patterns of species diversity are presented at the local scale of fjord basins or shelf stations, at the scale of entire fjords or shelf stations, and at the regional scale (pooled fjords versus pooled shelf stations). We first consider species diversity as measured by Shannon and Hurlbert rarefaction indices, and then discuss the two components of species diversity, species richness and evenness, separately because they offer different insights into ecological and evolutionary processes [40].

#### Local Scale

Both Shannon diversity (H′) and Hurlbert rarefaction diversity (Es(100)) were generally lower in fjord basins than at open shelf stations, although there was some fjord-shelf overlap (e.g., Inner Barilari Bay had H′ values similar to open shelf stations) (Figures 3C–F). This reduction in H′ and Es(100) in fjords appeared to be driven completely by lower species evenness because mean species densities (number of species per 90 m^2^ phototransect) were higher in fjords (Figures 3G & H), and Pielou's Evenness (J′) exhibited a pattern very similar to H′ and Es(100), i.e., generally lower evenness in fjords than at open shelf stations, with some fjord-shelf overlap (Figures 3I & J). Although H′, Es(100), species density and J′ exhibited substantial variability between fjord basins, none showed any tendency to decrease from outer to inner basins, or with proximity to glacial termini, even at distances of 2.5–6.5 km from termini (Figures 3C–J).

Ugland species-accumulation curves indicated that new species continued to accumulate at our sampling effort for all fjord basins and shelf stations (Figure S4), so we used species richness estimators to make comparisons of total species richness at the local scale (i.e., fjord basin and open shelf station). In contrast to H′, Es(100) and J′, Chao 1 species richness in fjord basins generally matched that on the open shelf (Figures 3K & L). In addition, there was no decrease in estimated total species richness with proximity to tidewater glaciers, with Chao 1 remaining stable within a fjord even at distances of 2.5–6.5 km from glacial termini (Figure 3K). Bootstrap and Jackknife 2 species richness estimators showed patterns essentially similar to Chao 1 in close proximity to glacial termini, with slightly higher total species richness estimates for shelf stations B and E compared to fjords (Figure S5).

#### Fjord Scale

Statistically significant differences in biodiversity metrics between fjords versus open shelf stations were evident when comparisons were made between individual fjords and the open shelf as a whole (Table 1). Results were more variable when the relative differences between individual fjords and individual shelf stations were analyzed, where results varied between sites and the metrics being tested. However, statistically significant differences were generally more common when open shelf stations were compared with Andvord and Flandres Bays (Table 1).

**Figure 5:**
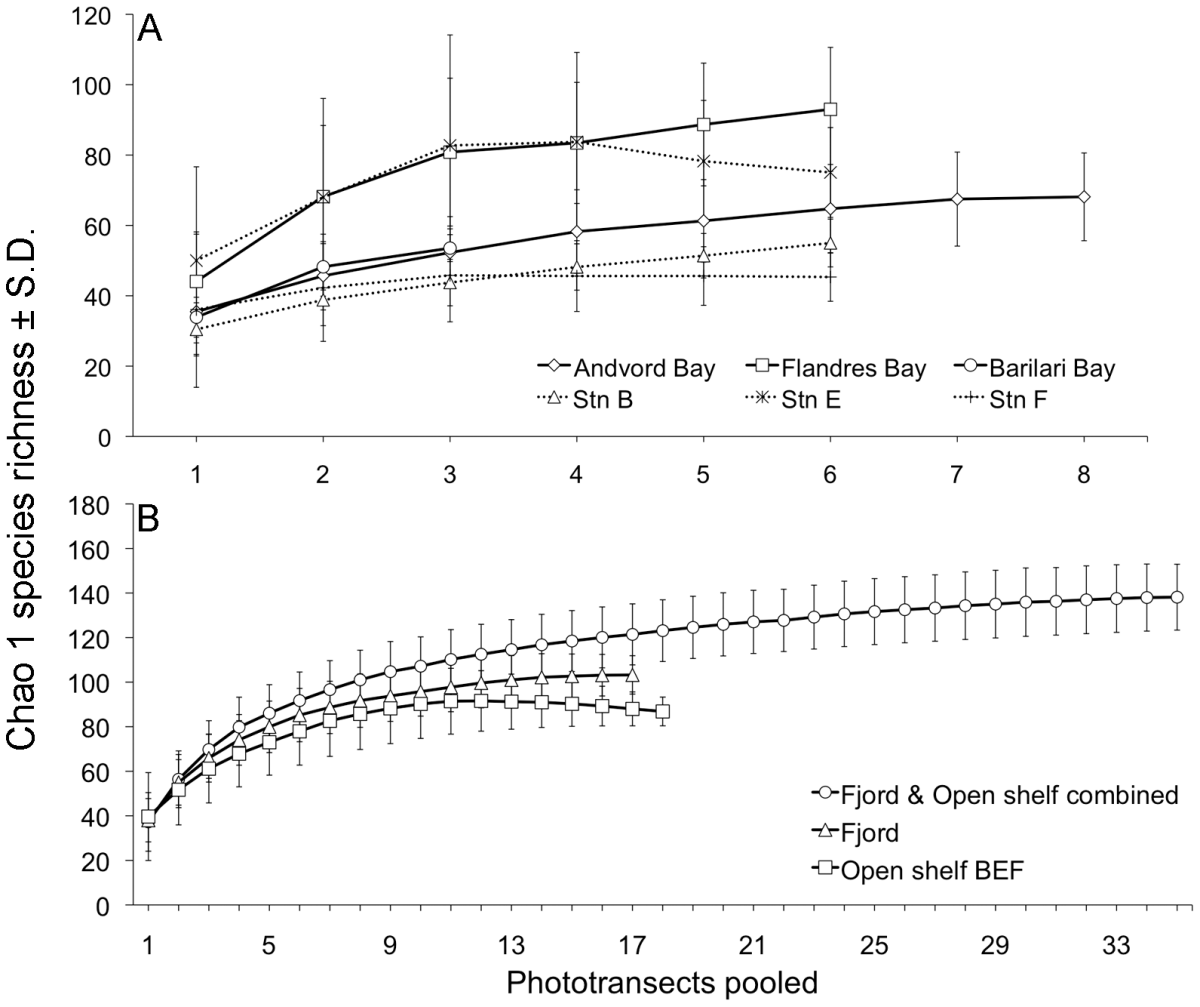
Patterns of estimated total epibenthic megafaunal species richness at the fjord and regional scale. (**A**) Estimated megafaunal species richness (Chao 1 ± 1 SD) at the fjord scale of whole fjords or open shelf stations, with increasing number of phototransects. (**B**) Estimated megafaunal species richness (Chao 1 ± 1 SD) at the regional scale of pooled fjord stations (triangles), pooled open shelf stations (squares) and fjord+open shelf stations combined (circles), with increasing number of phototransects.

Species accumulation curves for whole fjords and shelf stations also failed to approach asymptotes, indicating that species were still accumulating at the intensity of our sampling (150–400 photographs) for each of these soft sediment habitats (Figure S6). Estimated species richness, based on Chao 1, indicated that Andvord, Flandres and Barilari Bays had total species richness levels comparable to, or higher than, the open shelf (especially in the case of open shelf stations B and F), with Flandres Bay having the highest estimated species richness (Figure 5A). Statistically significant fjord-shelf differences were recorded for all fjords when compared to the open shelf as a whole, although no pairwise comparison was significant after Bonferroni correction (Table 1). Bootstrap and Jackknife 2 species richness estimators gave very similar results to Chao 1 (Figure S7); however there was no statistically significant difference between habitats (Table S10).

#### Regional Scale

At the regional scale (i.e., pooled fjords versus pooled shelf stations), fjords harbored both higher species richness and a substantial number of unique species compared to the open shelf. For both the pooled fjords and pooled shelf stations, Ugland species accumulation curves were beginning to level off, with fjords accumulating 14 (or 18%) more species than the open shelf (Figure S8). Estimated total species richness, using Chao 1, was also greater for fjords, with 16 more species than the open shelf (Figure 5B). In addition to greater species richness, pooled fjords accumulated 38 species absent from the open shelf stations, and the curve for accumulation of unique fjord species did not approach an asymptote, suggesting that more unique fjord species would be encountered with additional sampling (Figure S9). Chao 1 species richness for the entire region (i.e., for fjord and shelf transects combined) exceeded that for the pooled shelf stations by 52 species (S.D. = 14.8,) or ∼60%, indicating that fjords contributed substantial beta diversity, enhancing gamma diversity, for soft-sediment habitats at depths of ∼400–700 m on WAP margin (Figure 5B). Bootstrap and Jackknife 2 species richness estimators gave essentially the same results at the regional scale as Chao 1 (Figure S10). This fjord enhancement of beta diversity, as indicated by species richness estimators, is consistent with the nMDS and ANOSIM analyses presented above, which indicate that each fjord contains a distinct megafaunal assemblage.

### (a) Nekton and Drift Macroalgae

**Figure 6:**
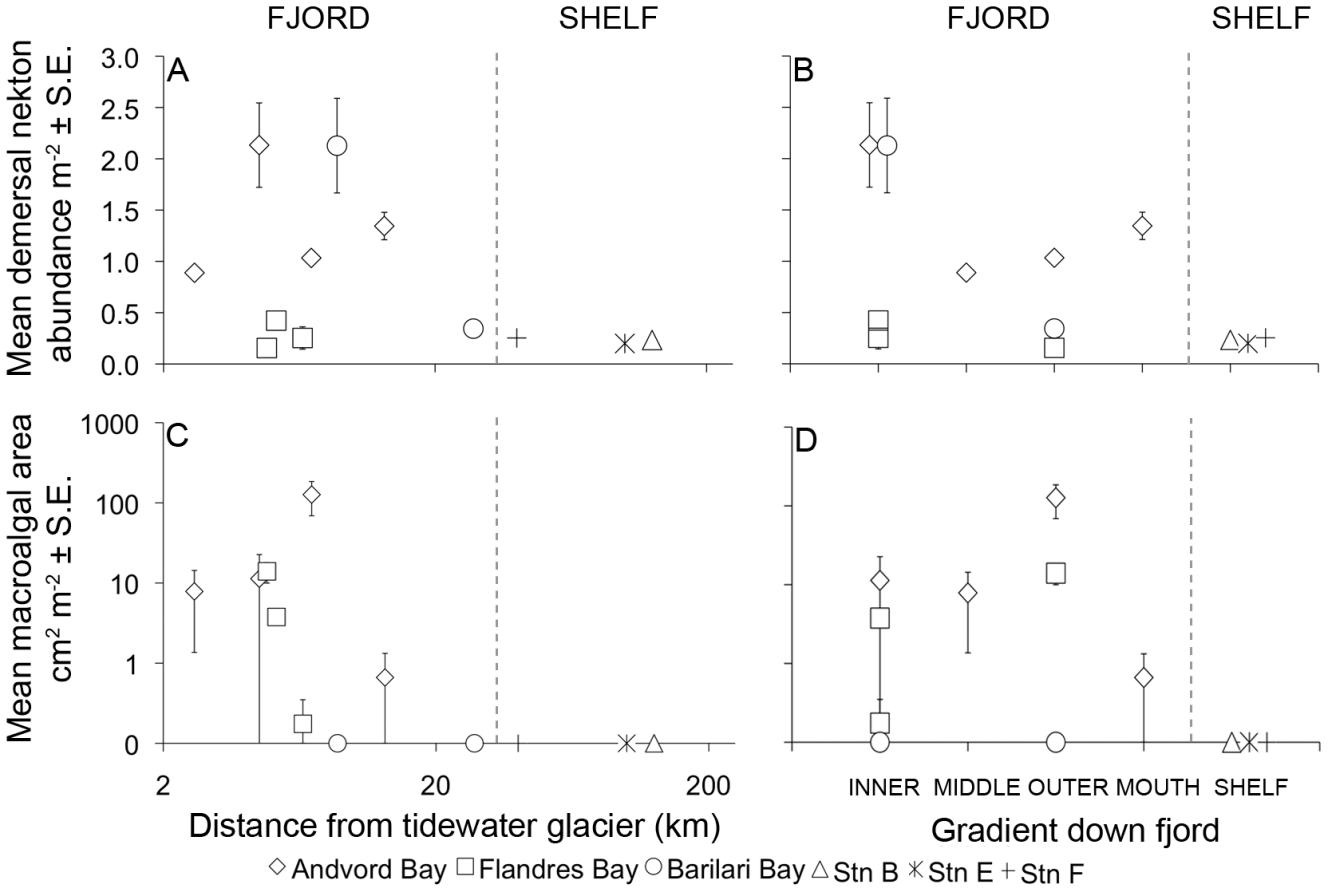
Demersal nekton abundance and area of macroalgal detritus at the local scale. Data are plotted as a function of distance to the nearest tidewater glacier, and position in basins down fjord. (**A–B**) Demersal nekton abundance (m^−2^) and (**C–D**) macroalgal detritus area (cm^2^ m^−2^) plotted for fjord basins and open shelf stations. Note that panels **C** and **D** have a log scale on the y-axis.

Demersal nekton abundances were 4 to 11-fold higher in Andvord and inner Barilari Bays than on the open shelf; the remaining fjord transects in Flandres and outer Barilari Bays had nekton abundances similar to, or slightly higher than, the open shelf (Figure 6A & B). The demersal nekton in Andvord, Flandres and Barilari Bays was dominated by krill, apparently *Euphausia superba* (Table S3). The trachymedusa *Ptychogastria polaris* was also very abundant in Andvord Bay and occurred at lower abundances in Flandres Bay (Table S3). On the open shelf, krill and a mysid were the most abundant nekton. The fjord nekton had higher taxonomic and species richness than the open shelf, with 3 phyla and six species identified in fjords, versus only 2 species of crustacean (krill and a mysid) recorded on the open shelf.

Drift macroalgae was very abundant in some fjord basins compared to the open shelf. While no drift algae were recorded on our shelf transects, all fjord stations except those within Barilari Bay contained drift algae (Figure 6C & D). Inner, middle and outer Andvord basins, and outer Flandres Bay, had substantial abundances of drift algae ranging from ∼8–130 cm^2^ m^−2^ (i.e., 0.08–1.3% cover). Drift algae could not be identified to species, but members of the macroalgae divisions Chlorophyta and Rhodophyceae were present in both Andvord and Flandres Bays.

### (b) Tests of Hypotheses

Megafaunal community patterns in the WAP fjords were inconsistent with our Hypotheses 1 and 2 derived from models of subpolar Arctic fjords. Faunal abundances were highly elevated in the WAP fjords, species richness and trophic complexity were comparable to levels on the open WAP shelf, and mobile deposit feeders constituted a small proportion (<20%) of the megafauna in the inner and middle basins of all three fjords; these results disprove nearly all components of Hypothesis 1. Shannon diversity (H′) and Hurlbert rarefaction diversity (Es(100)) were somewhat lower in some fjord basins than at some shelf stations but these differences were driven entirely by species evenness, not species richness. In addition, none of these parameters (i.e., faunal abundance, species richness, species diversity or trophic complexity) showed any increasing trend down fjord or with distance from glacial termini, wholly disproving Hypothesis 2. In summary, megafaunal community patterns in WAP subpolar fjords hosting tidewater glaciers are markedly different from expectations based on studies and conceptual models from Arctic subpolar fjords, where benthic communities appear to be heavily modified by burial disturbance [10], [29].

## Discussion

In contrast to expectations from Arctic fjord models [4], [5], [10], [13], soft-sediment epibenthic megafaunal communities in the subpolar fjords along the WAP were characterized by very high abundance relative to the adjacent open shelf at similar depths (∼400–700 m). The WAP fjord mean megafaunal abundances (9.5–43.2 m^−2^) also generally exceeded those at similar depths measured with similar image resolution around Antarctica in the Bellingshausen, Weddell, and Ross Seas, except at the mouth of Potter Cove, another Antarctic fjord [42], [43], [44], [45], [46] (Table 4). WAP fjord megafaunal densities were also very high compared to similar depths in Arctic subpolar fjords (2 to 42-fold higher [5], [27]) (Table 4) and to continental margins generally, exceeding megafaunal abundances (∼0.006–6 m^−2^) at all stations between 200–750 m depths reported in the global tabulation of Rex et al. [47]. WAP fjord megafaunal abundances also generally exceeded the abundance of epibenthic megafauna in Kaikoura Canyon on the eastern New Zealand margin, a bathyal site recognized as an intense hotspot of megafaunal standing crop [48]. Thus, by the standards of the both Antarctic shelf and continental margins generally, the WAP fjord basins must be considered to harbor remarkably high epibenthic megafaunal abundance.

**Table 4:** Epibenthic megafaunal abundances measured at similar depths to our study (400–750 m) in Antarctic and Arctic regions.

Location	Mean depth (m)	Data collection method	Mean megafaunal abundance (m^−2^)	Reference
WAP fjords (Andvord, Flandres and Barilari Bays)	436–725	Phototransects	9.5–43.2	This study
West Ross Sea	581–808	Video transects	10.7–13.3	Barry et al. [42]
King George Island, South Shetland Islands	430–750	Very high resolution (2 mm) photographic surveys*	30.8–56.2**	Piepenburg et al. [43]
Weddell Sea & Bellingshausen Seas	415–479	Video transects	5.9–12.7	Starmans et al. [44]
WAP shelf	526–641	Video transects	0.20–1.52	Sumida et al. [45]
Fimbul Ice Shelf region, Weddell Sea	510	Phototransects	0.89	Jones et al. [46]
Baffin Island Fjords, Arctic	424–750	Phototransects	1.04–3.71	Syvitski et al. [5]
Greenland Fjord Mouth	481–722	Phototransects	4.11–5.12	Jones et al. [27]

[43] photographs allowed counting of much smaller animals than in this study, making their results not strictly comparable to ours. The much higher resolution of the Piepenburg et al.

Mouth of Potter Cove fjord, King George Island.

High megafaunal abundances in the deep basins of WAP fjords suggest enhanced food inputs to these detritus-based ecosystems. The elevated abundance of demersal mysids and krill (which are also likely to be detritivores) in Andvord and Barilari Bays further suggests greater food availability. Enhanced detrital food flux in fjords relative to the open shelf could come from several sources. (1) The WAP subpolar fjords appear to experience more sustained phytoplankton blooms than the open shelf, with blooms extending well into the fall season ([22], M. Vernet pers. comm., C. Smith pers. obs). These sustained blooms may be a consequence of weak meltwater inflow, which adds nutrients and promotes stratification without creating high turbidity from glacial sediment loading [2], [3], [22]. (2) The WAP fjord basins may sustain a significant flux of macroalgal detritus cascading down the steep fjord walls [16]. The frequent occurrence of macroalgal detritus in photosurveys in Andvord and Flandres Bays, and its complete absence from the open shelf stations (Figure 6C & D), are consistent with enhanced macroalgal inputs to fjord floors. (3) WAP fjords may sustain substantial spatial nutrient subsidies from the open ocean as a consequence of immigration of Antarctic krill (*E. superba*) and baleen whales into fjords during fall seasons ([22], C. Smith pers. obs.). Some of the largest aggregations of krill (e.g. 10–100 kg m^−2^, or ∼10,000–1,000,000 individuals m^−2^ of water column) and feeding humpback whales (*Megaptera novaeangliae*) observed in the Southern Ocean over the last 20 yr have been recorded recently in WAP fjords, including Andvord Bay ([22], M. Zhou pers. comm.). These aggregating krill accumulate most of their biomass during 3–7 year life spans feeding in the open ocean; thus, the sinking of exuviae and carcasses from the dense seasonal krill aggregations in fjords constitutes spatial detrital subsidies from large areas of the open ocean to the much smaller WAP fjord ecosystems. If only a small percentage of the krill molt and/or die when densely aggregated in the fjords, this could substantially increase detrital food availability and alter food-web dynamics at fjord floors [49], [50]. Immigrating humpback whales may also stimulate fjord primary productivity and phytodetrital flux by excreting nutrients derived from foraging outside fjords, and by vertically “pumping” nutrients from deep fjord waters into the euphotic zone [51]. In summary, a number of pathways appear to potentially enhance food availability and megafaunal abundance at the WAP fjord floors. However, the quantitative importance of these pathways to fjord benthic food webs cannot be assessed without additional studies of phytoplankton bloom dynamics, fluxes of krill carcasses and exuviae, and the structure of fjord benthic food webs (e.g., based on analyses of stable isotopes and biomarkers).

The distinct community structure, moderate to high species richness, and substantial list of unique species in fjords compared to similar depths on the open WAP shelf (estimated to be ∼52 unique species by Chao 1; Figure 5B) indicate that the fjords contribute substantially to beta and gamma diversity in soft sediments at depths of ∼400–700 m in the WAP region. The fact that each fjord contained a different, essentially fjord-wide, community (Figure 4A) also suggests that each fjord either had markedly different habitat conditions, which seems unlikely, or that faunal exchange between Andvord, Flandres and Barilari Bays is restricted for some components of the megabenthos, such as species with direct development. The extraordinary abundance (>10 m^−2^) of pycnogonids in Flandres Bay compared to nearby Andvord Bay only 30 km away is consistent with limited inter-fjord exchange for species, such as pycnogonids, lacking planktonic dispersal [52], [53], [54]. However, the high abundance, large body size, and extraordinary fecundity of some fjord species with planktonic larvae, such as the ampharetid *A. membranifera* in Andvord Bay (Figure S2), also suggest that the fjords could be important larval sources for benthic populations on the broader WAP shelf if planktonic larvae can escape the fjords. The estuarine circulation characteristic of WAP fjords, with lower salinity surface waters flowing outward and bottom water flowing inward over fjord sills [3], may isolate populations remaining in bottom waters but export larvae rising to fjord surface waters out onto the open shelf. Fjord isolation of benthic populations unable to disperse into surface waters has been documented in New Zealand fjords with estuarine circulation [9]. The causes of community heterogeneity between the WAP fjords, including evaluation of the roles that fjords may play in isolating benthic populations or as sources of pelagic larvae, clearly merit further investigation.

Our observations of krill near the seafloor in both fjords and at open shelf stations are consistent with the speculation of Schmidt and colleagues [55] that krill may be foraging at the seabed. However, the krill we observed were rarely if ever in contact with the seafloor, we saw no swarms of krill near the seafloor, and the abundances of demersal krill (0.01–1.96 m^−2^) were very low compared to potential abundances in the water column above, e.g. up to 10,000–1,000,000 individuals m^−2^ in Andvord Bay ([22], M. Zhou pers. comm.). Thus, while our data suggest that krill may venture to the seafloor within the fjords and along the WAP shelf, our data do not suggest that a substantial proportion of the krill population was feeding at the seafloor during our times of study (the summers of 2008, 2009 and 2010, and winter of 2008). Further synchronous time-series studies of the water column and the seafloor are required to quantitatively evaluate the importance of krill foraging at the seabed.

Benthic community models from Arctic subpolar fjords, in which faunal abundance, species richness and diversity, and trophic complexity decline from the open shelf to inner fjords [10], [29], proved poor in predicting community structure in Andvord, Flandres and Barilari Bays. For example, Włodarska-Kowalczuk et al. [10], [29] document dramatic declines in macrobenthic species richness, evenness and functional diversity from the open shelf to inner subpolar fjords in the Arctic, and ascribe these changes to environmental stress related to glacial processes (e.g., high turbidity and sedimentation) in inner fjords. In contrast, our data from WAP subpolar fjords show little evidence of increasing environmental stress up fjords, with enhanced species richness, no decline in functional complexity, and at most modest declines in species evenness (and total diversity) from the open shelf to inner fjord basins. The modest declines in species evenness in WAP subpolar fjords are most likely a consequence of enhanced productivity rather than greater environmental stress, since environmental stress typically reduces both species richness and evenness [29], [40].

Why are benthic communities in WAP subpolar fjords so different from those in the Arctic? We hypothesize that the differences result from much weaker meltwater influence, and much less disturbance from terrigenous sedimentation, in WAP fjords compared to the Arctic because these subpolar Antarctic fjords are in earlier stages of climate warming. Although the WAP fjords sustain substantial inputs of glacial ice, this is accompanied by relatively little glacial meltwater and sediment inputs, leading to low turbidity levels in surface waters and moderate sedimentation rates (0.5–2 cm y^−1^) even within 5 km of glacial termini [3], [20]. Thus, the WAP fjords appear to experience little inhibition of phytoplankton production from water column turbidity, as well as limited burial disturbance at basin floors. In contrast, the subpolar Arctic fjords are heavily influenced by meltwater and terrigenous sediment loading, sustaining high turbidity levels and very high sedimentation rates (e.g., 2–25 cm y^−1^), which limit primary production and select for species and functional groups (small bodied, mobile, surface deposit feeders) adapted to burial stress [3], [5], [6], [8], [10], [13], [28], [29]. As the WAP continues to warm, the fluxes of meltwater and terrigenous sediments into WAP fjords will increase [3], [6], [20], [56], very likely reducing primary production in the water column, increasing seafloor burial disturbance, and potentially reducing the standing crop, diversity, and trophic complexity of the WAP fjord macro- and megabenthos. The observations of high burial disturbance and consequent low macrobenthic abundance and diversity in Eczurra Inlet, Admiralty Bay [14], [15], [16], a substantially warmer Antarctic fjord [20], are highly consistent with these predictions. Thus, we hypothesize that the extraordinary ecosystems in WAP fjords, which provide habitat and foraging areas for krill and baleen whales [22] and constitute hotspots of benthic community abundance and beta diversity, will be deleteriously impacted by the very rapid climate warming occurring along the Antarctic Peninsula [21], [31], [32], [56], [57], [58], [59]. Because such productivity/biodiversity hotspots can play disproportionate roles in the feeding and recruitment of mobile species (e.g., krill, baleen whales, juvenile fish) [22], [23], [48], [50], [60] and in maintaining biodiversity in heterogeneous ecological landscapes [61], [62], we suggest that there is an urgent need to develop a better understanding of the structure, function and climate sensitivity of these WAP subpolar fjord ecosystems.

## Supporting Information

Figure S1 **Yoyo Camera system.** Yoyo Camera system used for photographic surveys in fjord basins and at open shelf stations. This system consists of a tubular steel frame supporting an Ocean Imaging Systems DSC 10000 digital still camera in titanium housing (10.2 megapixel, 20-mm, Nikon D-80 Camera), with an Ocean Imaging Systems 3831 Strobe (200 W-S) located 1-m from the camera at an angle of 26° from vertical, and a Model 494 Bottom Contact Switch.
(TIF)

Figure S2 **Ampharetid polychaete species typical of fjord and open shelf habitats.** (**A**) Example of a typical open shelf ampharetid polychaete, *Amphicteis* sp. (**B–C**) Typical fjord ampharetids from Andvord Bay. Reproductively ripe (**B**) female and (**C**) male *Amythas membranifera*, with eggs or sperm visible in the body cavity.
(TIF)

Figure S3 **Dendrogram of epibenthic megafaunal community structure in fjord and open shelf habitats.** Community-structure results based on cluster analysis (using Bray-Curtis similarity and average linkage) of the epibenthic megafaunal assemblages from fjord basins and open shelf stations. See Figure 4A for corresponding non-metric multidimensional scaling (nMDS) plot, including a description of open shelf station annotations.
(TIF)

Figure S4 **Mean epibenthic megafaunal species accumulated within fjord basins or shelf stations.** Mean accumulation of species at the local scale of fjord basin or open shelf station for all epibenthic megafaunal species in (**A**) Andvord, (**B**) Flandres and (**C**) Barilari Bays, and open shelf stations B, E and F. Basins: AI = Andvord Bay inner; AM = Andvord Bay middle; AO = Andvord Bay outer; AMTH = Andvord Bay mouth; FIA = Flandres Bay inner A; FIB = Flandres Bay inner B; FO = Flandres Bay outer; BI = Barilari Bay inner; and BO = Barilari Bay outer.
(TIF)

Figure S5 **Estimated total species richness using Bootstrap and Jackknife 2 richness estimators at the local scale.** Data are plotted as a function of distance to the nearest tidewater glacier, and position in basins down fjord. (**A–B**) Bootstrap species richness and (**C–D**) Jackknife 2 species richness for fjord basins and open shelf stations.
(TIF)

Figure S6 **Mean epibenthic megafaunal species accumulated at the fjord scale.** Mean accumulation of species at the fjord scale of whole fjords or open shelf stations for all epibenthic megafaunal species in Andvord, Flandres and Barilari Bays, and open shelf stations B, E and F, with increasing number of phototransects.
(TIF)

Figure S7 **Estimated total species richness using Bootstrap and Jackknife 2 richness estimators accumulated at the fjord scale.** Accumulation of total species richness at the scale of whole fjords or open shelf stations for all epibenthic megafaunal species in Andvord, Flandres and Barilari Bays, and open shelf stations B, E and F using (**A**) Bootstrap species richness and (**B**) Jackknife 2 species richness estimators, with increasing number of phototransects.
(TIF)

Figure S8 **Mean epibenthic megafaunal species accumulated at the regional scale.** Mean accumulation of species at the regional scale of pooled fjord stations (triangles), pooled open shelf stations (squares), and fjord+open shelf stations combined (circles) for all epibenthic megafaunal species, with increasing number of phototransects.
(TIF)

Figure S9 **Mean accumulation of unique epibenthic megafaunal species.** Mean accumulation of species unique to fjords and open shelf stations with increasing number of phototransects. ‘Unique’ fjord or open shelf species are those epibenthic megafaunal species only observed in fjord or open shelf habitats, respectively.
(TIF)

Figure S10 **Estimated total species richness using Bootstrap and Jackknife 2 richness estimators accumulated at the regional scale.** Accumulation of total species richness at the regional scale of pooled fjord stations (triangles), pooled open shelf stations (squares), and fjord+open shelf stations combined (circles) using (**A**) Bootstrap species richness and (**B**) Jackknife 2 species richness estimators, with increasing number of phototransects.
(TIF)

Table S1 **Arctic fjord studies.** Arctic fjord studies indicating substantial burial disturbance of inner – middle fjord benthos, and faunal component(s) for which these effects were documented.
(DOC)

Table S2 **Station locations.** Seafloor photosurveys for this study taken at ten stations in Andvord, Flandres and Barilari Bays during NBP10-01 (2010) from RVIB N. B. Palmer, and at the three open shelf stations B, E and F during three cruises aboard the ASRV L. M. Gould and RVIB N.B. Palmer in 2008 and 2009 (LMG08-02 = summer (1); NBP08-08 = winter (2); and LMG09-02 = summer (3)).
(DOC)

Table S3 **Dominant demersal nekton species by percentage of total abundance in WAP fjord basins and at open shelf stations.** Data are mean abundances m^−2^ ± 1 SE using phototransects as replicates. Basins: AI = Andvord Bay inner; AM = Andvord Bay middle; AO = Andvord Bay outer; AMTH = Andvord Bay mouth; FIA = Flandres Bay inner A; FIB = Flandres Bay inner B; FO = Flandres Bay outer; BI = Barilari Bay inner; and BO = Barilari Bay outer.
(DOC)

Table S4 **ANOSIM analysis.** ANOSIM pairwise tests between individual fjords and open shelf stations.
(DOC)

Table S5 **SIMPER analysis of fjords and open shelf stations.** Av.Abund: based on 4^th^ root transformed data, Av.Sim = average of the bray curtis similarities between all pairs of sites; Sim/SD = ratio of average contribution (column 2) divided by SD of those contributions across all pairs of samples making up this average - larger number means more consistently contributes to similarity between sites; Contrib% = percentage contribution of total percentage average similarity e.g. 71.20 Andvord Bay; and Cum.% = culminated % contributions in column 4 until cut off % (in this case ∼50%).
(DOC)

Table S6 **SIMPER analysis of Andvord Bay versus open shelf stations.** Av.Abund = based on 4^th^ root transformed data; Av.Diss = average of the bray curtis dissimilarities between all pairs of sites; Diss/SD = ratio of average contribution (column 2) divided by SD of those contributions across all pairs of samples making up this average - larger number means more consistently contributes to dissimilarity between sites; Contrib% = percentage contribution of total percentage average dissimilarity e.g. 88.0 Andvord Bay & B; and Cum.% = culminated % contributions in column 5 until cut off % (in this case ∼50%).
(DOC)

Table S7 **SIMPER analysis of Flandres Bay versus open shelf stations.** Av.Abund = based on 4^th^ root transformed data; Av.Diss = average of the bray curtis dissimilarities between all pairs of sites; Diss/SD = ratio of average contribution (column 2) divided by SD of those contributions across all pairs of samples making up this average - larger number means more consistently contributes to dissimilarity between sites; Contrib% = percentage contribution of total percentage average dissimilarity e.g. 81.9 Flandres Bay & B; and Cum.% = culminated % contributions in column 5 until cut of % (in this case ∼50%).
(DOC)

Table S8 **SIMPER analysis of Barilari Bay versus open shelf stations.** Av.Abund = based on 4^th^ root transformed data; Av.Diss = average of the bray curtis dissimilarities between all pairs of sites; Diss/SD = ratio of average contribution (column 2) divided by SD of those contributions across all pairs of samples making up this average - larger number means more consistently contributes to dissimilarity between sites; Contrib% = percentage contribution of total percentage average dissimilarity e.g. 82.3 Barilari & B; and Cum.% = culminated % contributions in column 5 until cut of % (in this case ∼50%).
(DOC)

Table S9 **Shared and unique species inventory.** List of shared and unique species combining all WAP fjords and open shelf stations observed during this study. *Stylocordyla chupachups (Porifera: Hadromerida) previously reported as *S. borealis* (Lovén, 1868) in Uriz M-J, Gili J-M, Orejas C, Perez-Porro A-R (2011) Do bipolar distributions exist in marine sponges? *Stylocordyla chupachups* sp. nv. (Porifera: Hadromerida) from the Weddell Sea (Antarctic), previously reported as *S. borealis* (Lovén, 1868). Polar Biology 34: 243–255.
(DOC)

Table S10 **Differences in estimated total epibenthic megafaunal species richness between fjords and open shelf stations.** Higher values in the fjords are indicated by a “+”; lower values in the fjords by a “−”. Differences between fjords and the open shelf as a whole (Stations B, E and F) were tested using the Kruskal-Wallis test. Pairwise comparisons between individual fjords and individual open shelf stations were not addressed as no significant difference was identified between fjords versus the open shelf as a whole. ****P<0.0001, ***P<0.001, **P<0.01, *P<0.05 and N.S. (Non-significant) P>0.05.
(DOC)
