# Supplementary figures and images for: Megafaunal Communities in Rapidly Warming Fjords along the West Antarctic Peninsula: Hotspots of Abundance and Beta Diversity
Source: PLoS One. 2013 Dec 3;8(12):e77917. doi: 10.1371/journal.pone.0077917 (PMC3848936; doi:10.1371/journal.pone.0077917)

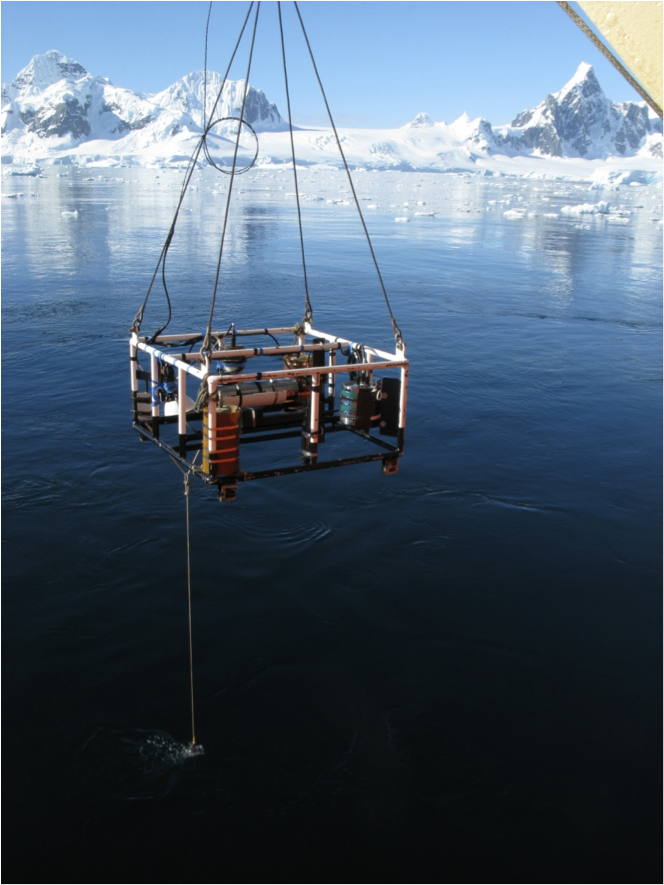

Supplement: Figure S1 — Yoyo Camera system. Yoyo Camera system used for photographic surveys in fjord basins and at open shelf stations. This system consists of a tubular steel frame supporting an Ocean Imaging Systems DSC 10000 digital still camera in titanium housing (10.2 megapixel, 20-mm, Nikon D-80 Camera), with an Ocean Imaging Systems 3831 Strobe (200 W-S) located 1-m from the camera at an angle of 26° from vertical, and a Model 494 Bottom Contact Switch. (TIF) [file pone.0077917.s001.tif]

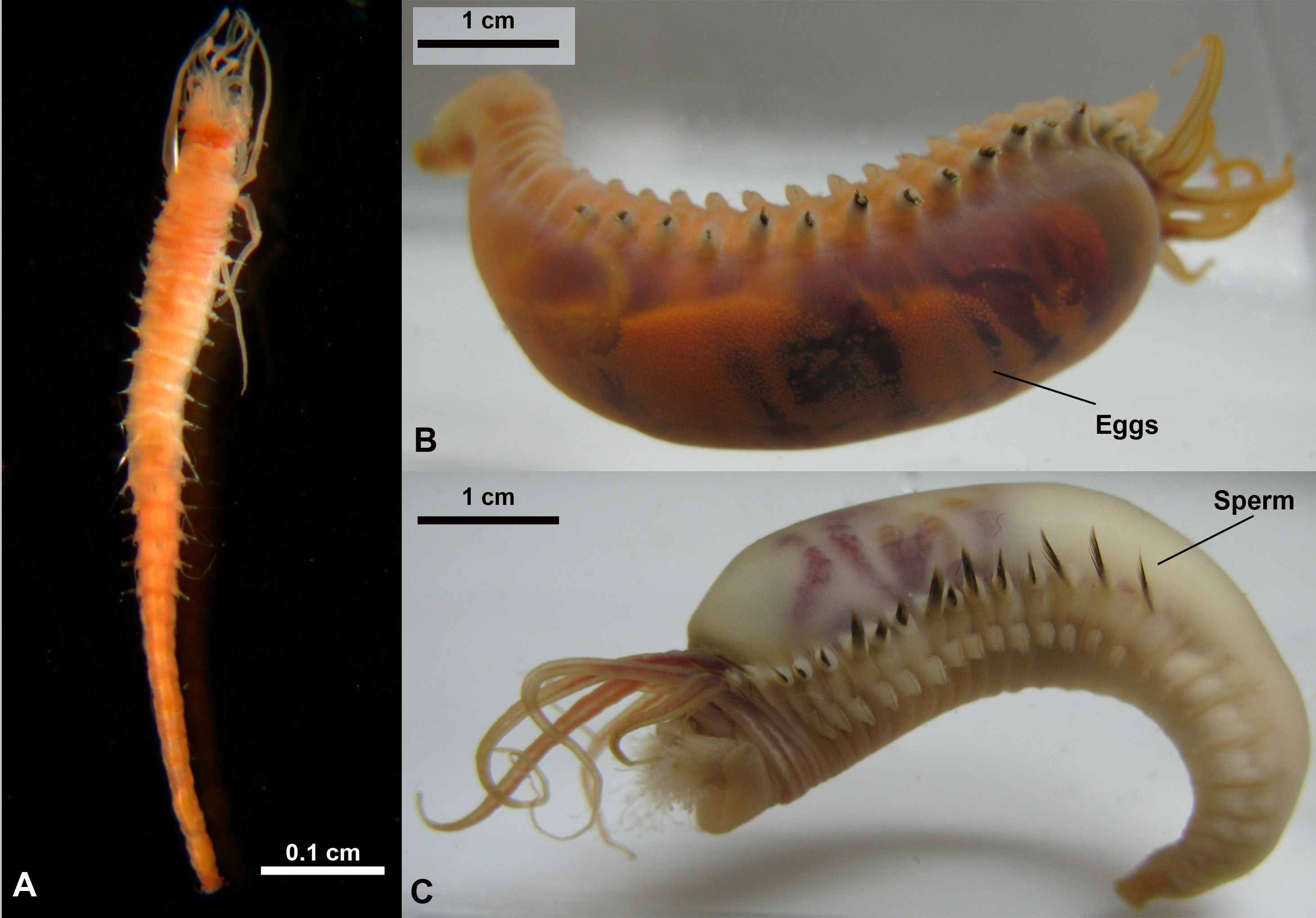

Supplement: Figure S2 — Ampharetid polychaete species typical of fjord and open shelf habitats. (A) Example of a typical open shelf ampharetid polychaete, Amphicteis sp. (B–C) Typical fjord ampharetids from Andvord Bay. Reproductively ripe (B) female and (C) male Amythas membranifera, with eggs or sperm visible in the body cavity. (TIF) [file pone.0077917.s002.tif]

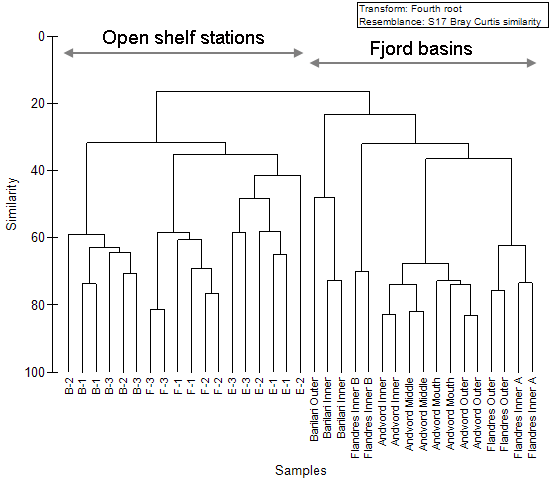

Supplement: Figure S3 — Dendrogram of epibenthic megafaunal community structure in fjord and open shelf habitats. Community-structure results based on cluster analysis (using Bray-Curtis similarity and average linkage) of the epibenthic megafaunal assemblages from fjord basins and open shelf stations. See Figure 4A for corresponding non-metric multidimensional scaling (nMDS) plot, including a description of open shelf station annotations. (TIF) [file pone.0077917.s003.tif]

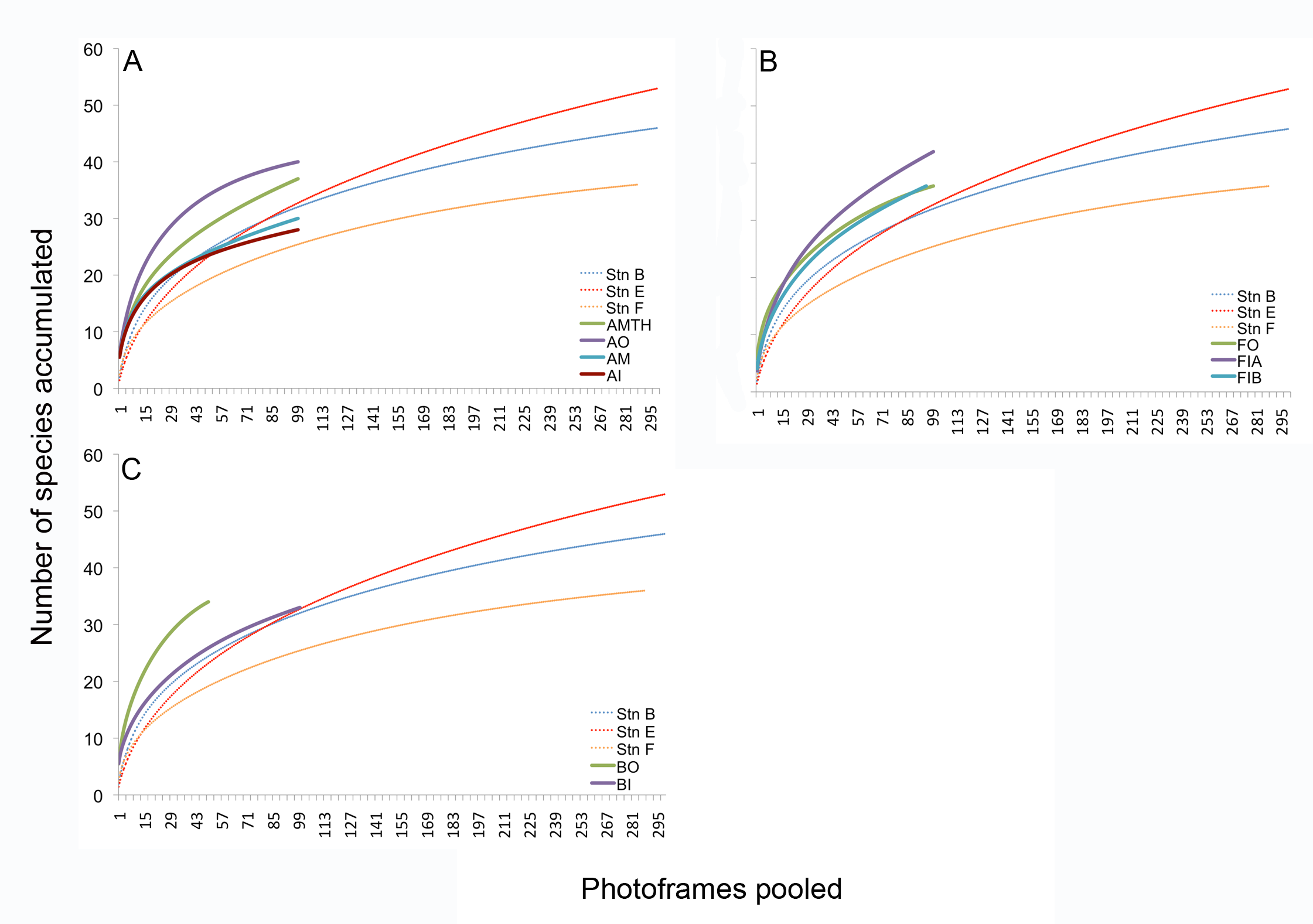

Supplement: Figure S4 — Mean epibenthic megafaunal species accumulated within fjord basins or shelf stations. Mean accumulation of species at the local scale of fjord basin or open shelf station for all epibenthic megafaunal species in (A) Andvord, (B) Flandres and (C) Barilari Bays, and open shelf stations B, E and F. Basins: AI = Andvord Bay inner; AM = Andvord Bay middle; AO = Andvord Bay outer; AMTH = Andvord Bay mouth; FIA = Flandres Bay inner A; FIB = Flandres Bay inner B; FO = Flandres Bay outer; BI = Barilari Bay inner; and BO = Barilari Bay outer. (TIF) [file pone.0077917.s004.tif]

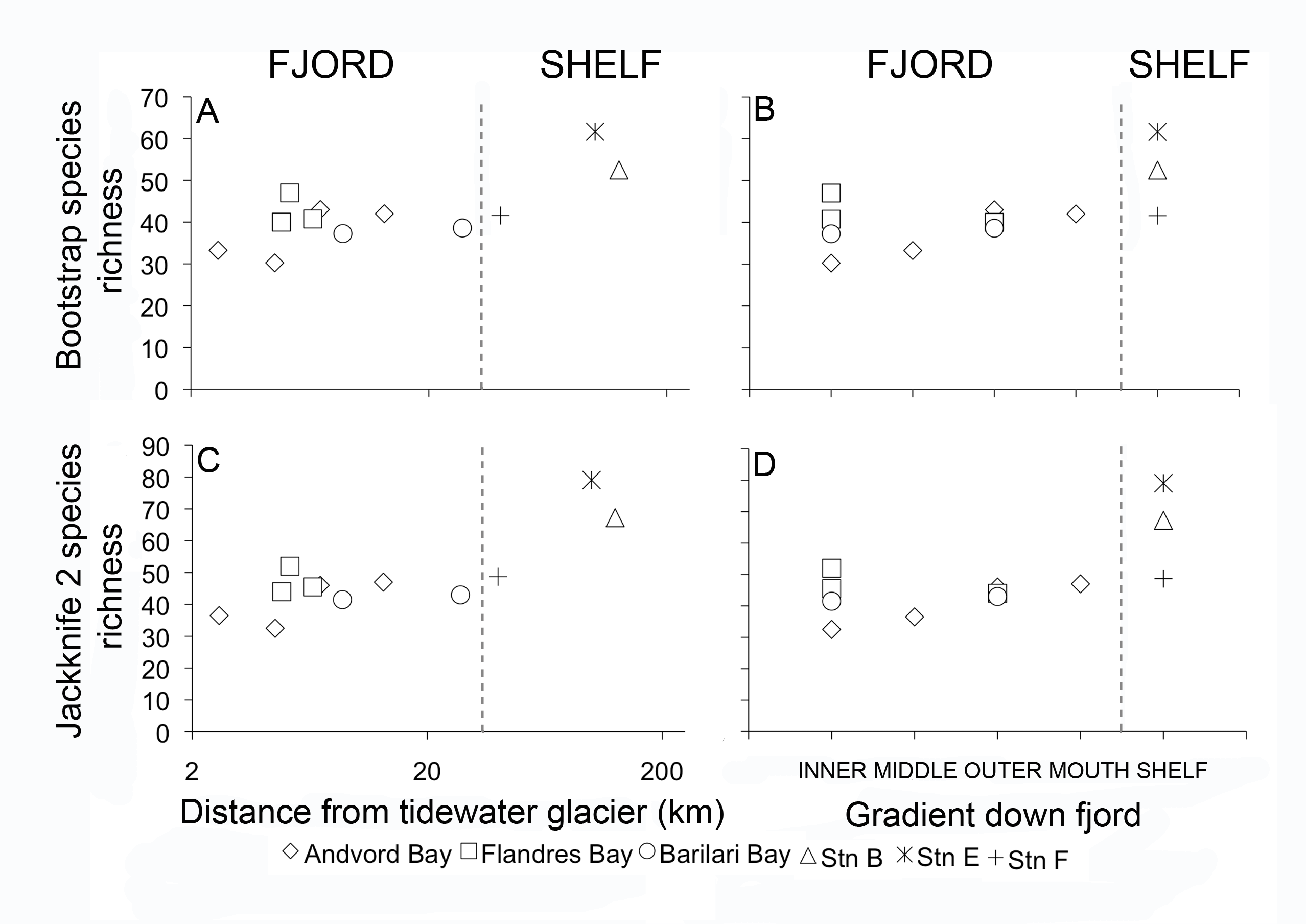

Supplement: Figure S5 — Estimated total species richness using Bootstrap and Jackknife 2 richness estimators at the local scale. Data are plotted as a function of distance to the nearest tidewater glacier, and position in basins down fjord. (A–B) Bootstrap species richness and (C–D) Jackknife 2 species richness for fjord basins and open shelf stations. (TIF) [file pone.0077917.s005.tif]

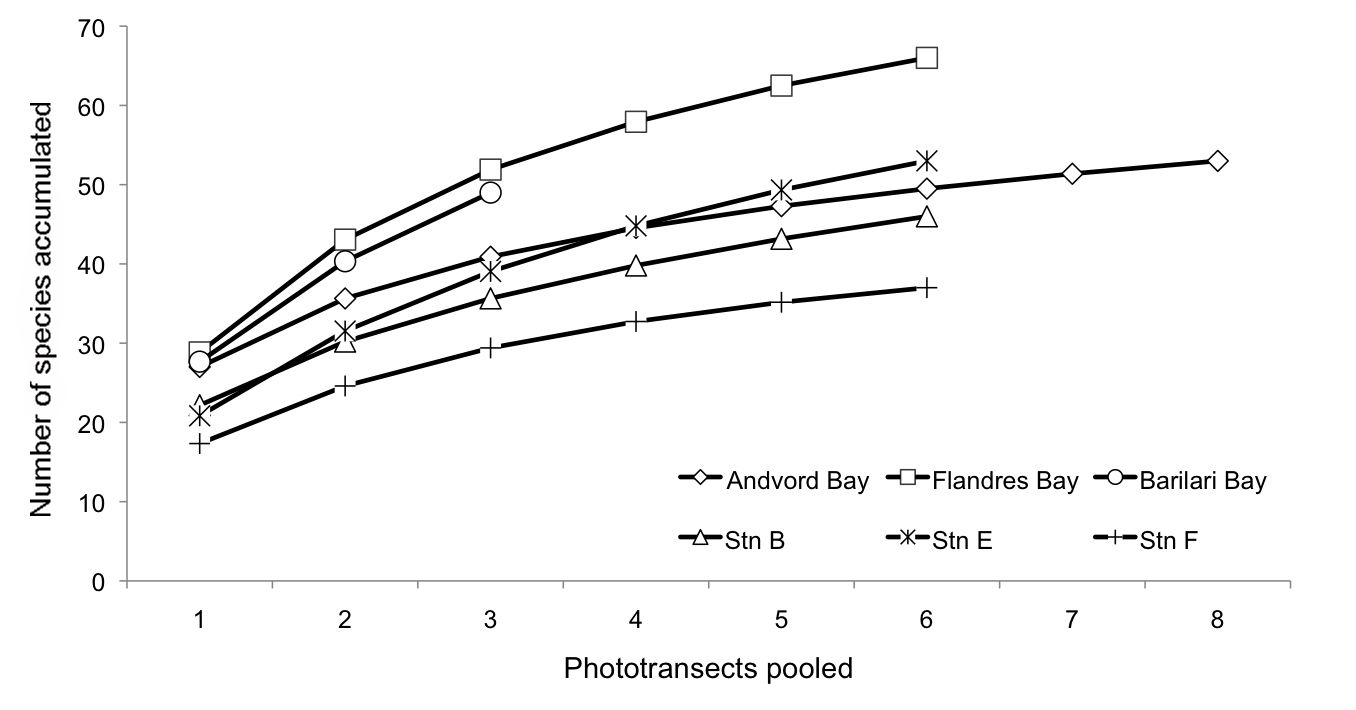

Supplement: Figure S6 — Mean epibenthic megafaunal species accumulated at the fjord scale. Mean accumulation of species at the fjord scale of whole fjords or open shelf stations for all epibenthic megafaunal species in Andvord, Flandres and Barilari Bays, and open shelf stations B, E and F, with increasing number of phototransects. (TIF) [file pone.0077917.s006.tif]

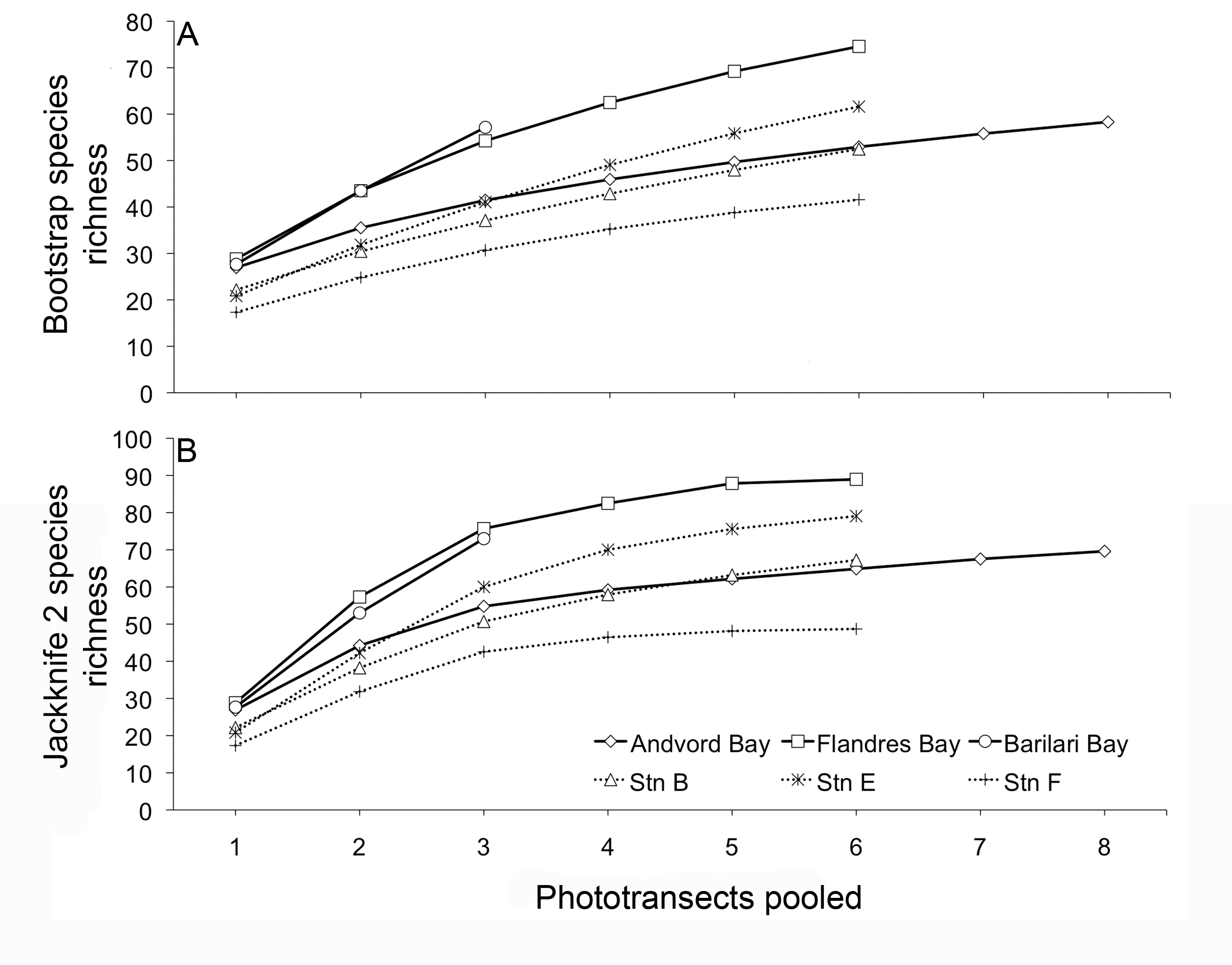

Supplement: Figure S7 — Estimated total species richness using Bootstrap and Jackknife 2 richness estimators accumulated at the fjord scale. Accumulation of total species richness at the scale of whole fjords or open shelf stations for all epibenthic megafaunal species in Andvord, Flandres and Barilari Bays, and open shelf stations B, E and F using (A) Bootstrap species richness and (B) Jackknife 2 species richness estimators, with increasing number of phototransects. (TIF) [file pone.0077917.s007.tif]

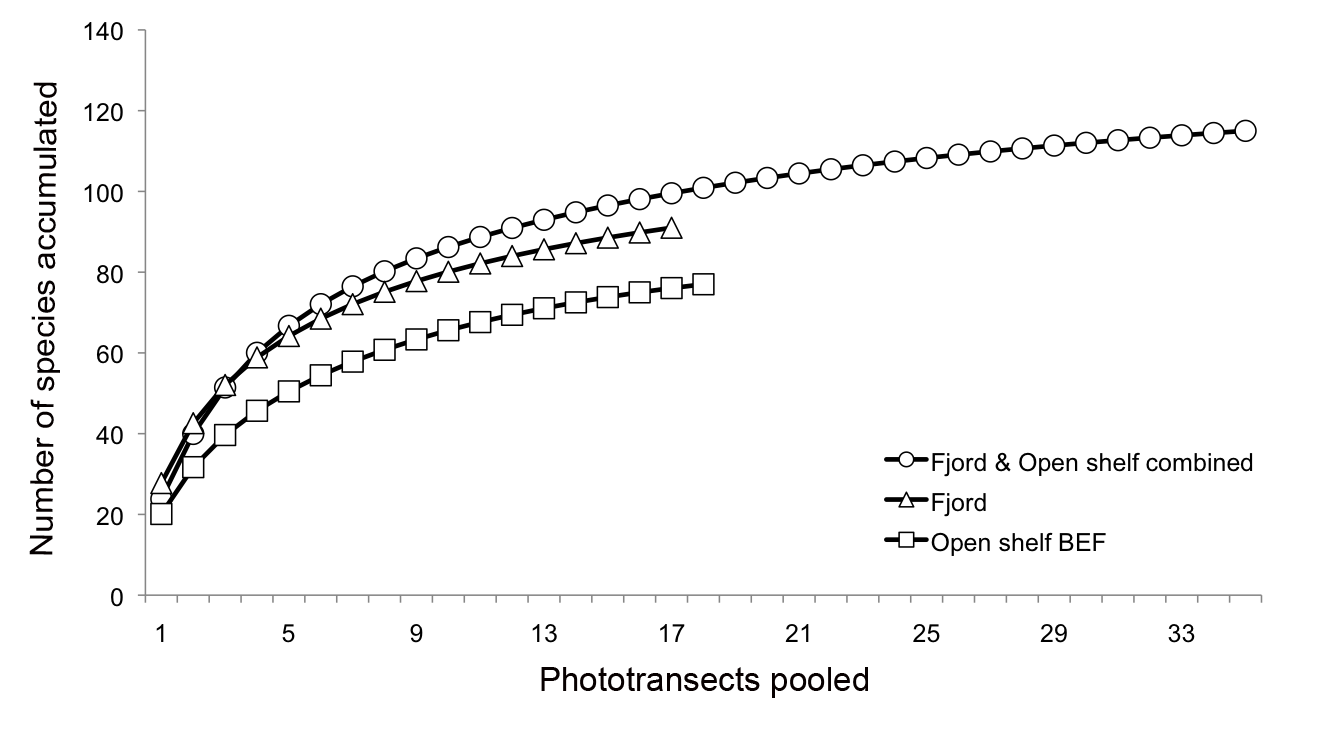

Supplement: Figure S8 — Mean epibenthic megafaunal species accumulated at the regional scale. Mean accumulation of species at the regional scale of pooled fjord stations (triangles), pooled open shelf stations (squares), and fjord+open shelf stations combined (circles) for all epibenthic megafaunal species, with increasing number of phototransects. (TIF) [file pone.0077917.s008.tif]

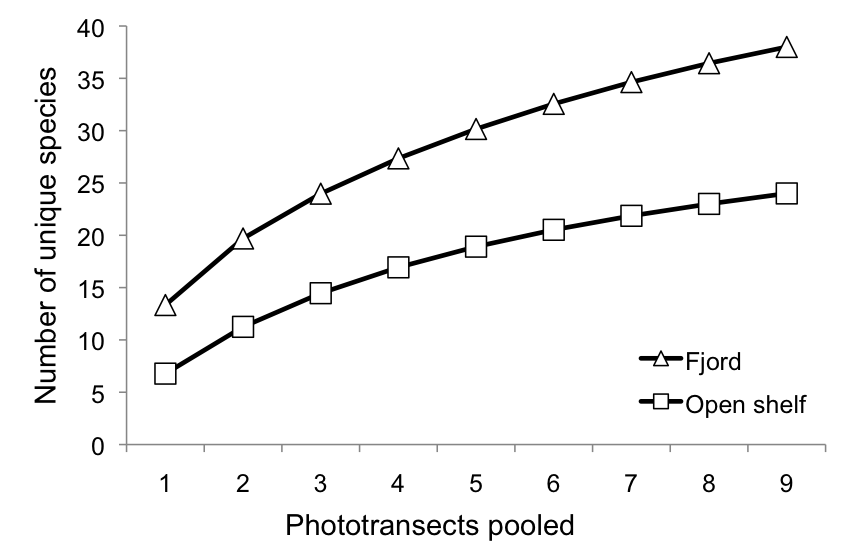

Supplement: Figure S9 — Mean accumulation of unique epibenthic megafaunal species. Mean accumulation of species unique to fjords and open shelf stations with increasing number of phototransects. ‘Unique’ fjord or open shelf species are those epibenthic megafaunal species only observed in fjord or open shelf habitats, respectively. (TIF) [file pone.0077917.s009.tif]

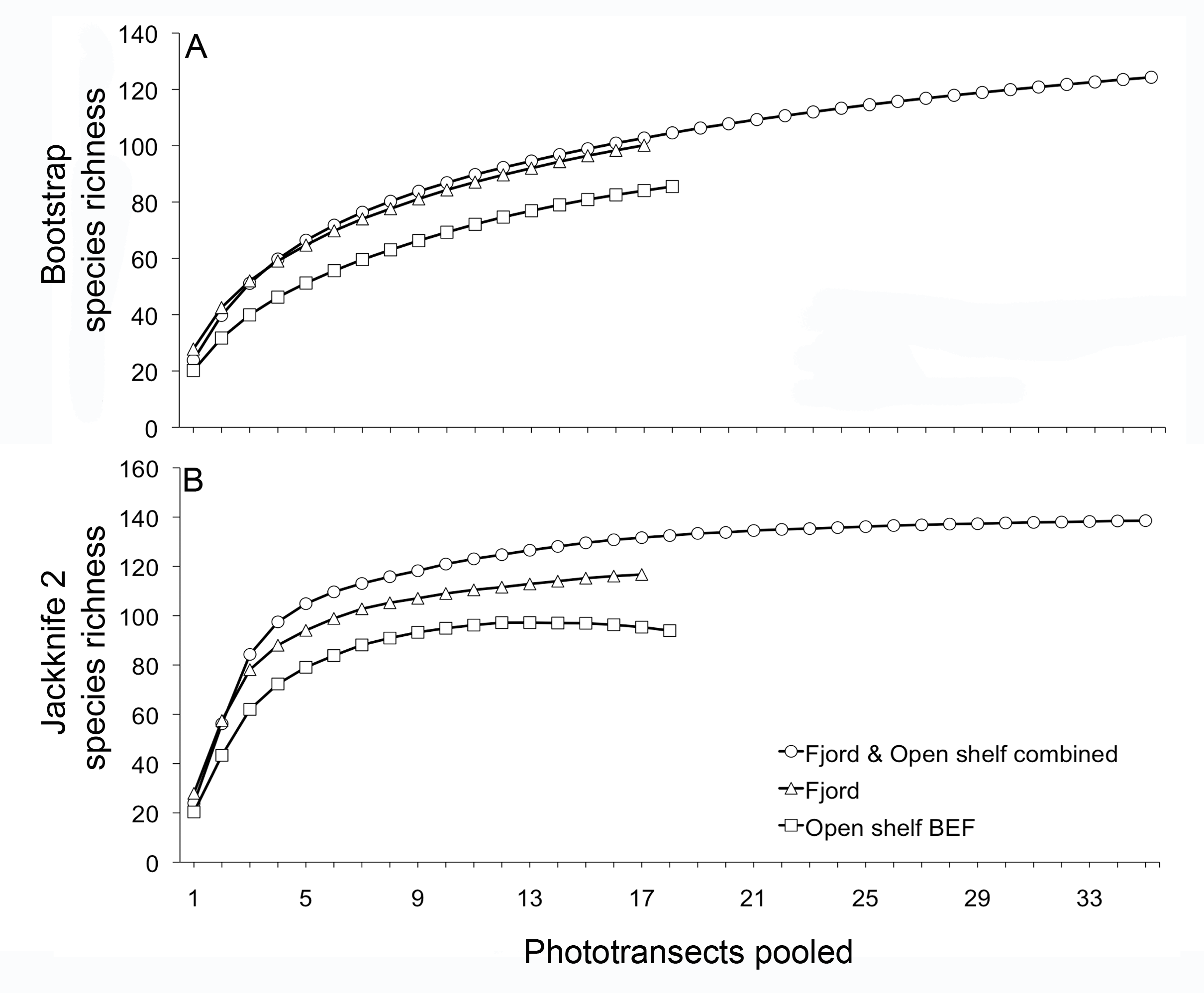

Supplement: Figure S10 — Estimated total species richness using Bootstrap and Jackknife 2 richness estimators accumulated at the regional scale. Accumulation of total species richness at the regional scale of pooled fjord stations (triangles), pooled open shelf stations (squares), and fjord+open shelf stations combined (circles) using (A) Bootstrap species richness and (B) Jackknife 2 species richness estimators, with increasing number of phototransects. (TIF) [file pone.0077917.s010.tif]
