# Supplementary material for: Megafaunal Communities in Rapidly Warming Fjords along the West Antarctic Peninsula: Hotspots of Abundance and Beta Diversity
Source: PLoS One. 2013 Dec 3;8(12):e77917. doi: 10.1371/journal.pone.0077917 (PMC3848936; doi:10.1371/journal.pone.0077917)
Supplement: Table S1 — Arctic fjord studies. Arctic fjord studies indicating substantial burial disturbance of inner – middle fjord benthos, and faunal component(s) for which these effects were documented. (DOC) [file pone.0077917.s011.doc]

| **Location** | **Benthic community studied** | **Reference** |
| --- | --- | --- |
| Kongsfjorden, Svalbard (high Arctic fjord) | Macrofauna | #4, Kedra et al. (2010) |
| Baffin Island Fjords (subarctic and arctic fjords) | Macrofauna and megafauna | #5, Syvitski et al. (1989) |
| Subarctic & arctic locations (review) | Macrofauna | #6, Wesławski et al. (2011) |
| Kongsfjorden, Svalbard (high Arctic fjord) | Macrofauna | #10, Włodarska-Kowalczuk et al. (2005) |
| Svalbard glacial bays (high Arctic) | Macrofauna | #11, Włodarska-Kowalczuk et al. (1998) |
| van Mijenfjord, Svalbard (high arctic fjord) | Macrofauna | #13, Renaud et al. (2007) |
| Norwegian Holandsfjord & two Svalbard fjords (the van Mijenfjord & the Raudfjord) | Macrofauna | #25, Holte & Gulliksen (1998) |
| Kongsfjord & van Mijenfjord, Svalbard (high Arctic fjords) | Macrofauna (polychaetes) | #26, Włodarska-Kowalczuk et al. (2007) |
| Kangerdlugssuaq Fjord, Arctic Greenland | Megafauna | #27, Jones et al. (2007) |
| Kongsfjorden, Svalbard (high Arctic fjord) | Macrofauna | #28, Fetzer et al. (2002) |
| Hornsund, van Mijenfjord & Kongsfjorden, Svalbard (high Arctic) | Macrofauna | #29, Włodarska-Kowalczuk et al. (2012) |
| Kongsfjorden, Svalbard (high Arctic) | Macrofauna | #30, Włodarska-Kowalczuk & Pearson (2004) |
