# Supplementary material for: Megafaunal Communities in Rapidly Warming Fjords along the West Antarctic Peninsula: Hotspots of Abundance and Beta Diversity
Source: PLoS One. 2013 Dec 3;8(12):e77917. doi: 10.1371/journal.pone.0077917 (PMC3848936; doi:10.1371/journal.pone.0077917)
Supplement: Table S2 — Station locations. Seafloor photosurveys for this study taken at ten stations in Andvord, Flandres and Barilari Bays during NBP10-01 (2010) from RVIB N. B. Palmer, and at the three open shelf stations B, E and F during three cruises aboard the ASRV L. M. Gould and RVIB N.B. Palmer in 2008 and 2009 (LMG08-02 = summer (1); NBP08-08 = winter (2); and LMG09-02 = summer (3)). (DOC) [file pone.0077917.s012.doc]

|  |  |  | **Coordinates (Degrees Lat. and Long.)** | | | |  |
| --- | --- | --- | --- | --- | --- | --- | --- |
| **Site** | **Station (basin or cruise)** | **Photo-transect (CRS #)** | **Transect start** | | **Transect end** | | **Mean depth (m)** |
| Andvord Bay | Mouth (AMTH) | 1289 | -64.780 | -62.875 | -64.778 | -62.864 | 533 |
|  |  | 1290 | -64.786 | -62.877 | -64.784 | -62.889 | 528 |
|  | Outer (AO) | 1283 | -64.782 | -62.744 | -64.780 | -62.728 | 551 |
|  |  | 1284 | -64.781 | -62.745 | -64.781 | -62.732 | 534 |
|  | Middle (AM) | 1337 | -64.824 | -62.649 | -64.819 | -62.659 | 436 |
|  |  | 1338 | -64.825 | -62.655 | -64.817 | -62.657 | 437 |
|  | Inner (AI) | 1285 | -64.858 | -62.563 | -64.863 | -62.559 | 523 |
|  |  | 1286 | -64.658 | -62.565 | -64.863 | -62.561 | 526 |
|  |  |  |  |  |  |  |  |
| Flandres Bay | Outer (FO) | 1281 | -65.002 | -63.326 | -65.003 | -63.313 | 725 |
|  |  | 1282 | -65.003 | -63.318 | -65.005 | -63.306 | 723 |
|  | Inner A (FIA) | 1279 | -65.054 | -63.114 | -65.053 | -63.100 | 686 |
|  |  | 1280 | -65.053 | -63.114 | -65.058 | -63.101 | 672 |
|  | Inner B (FIB) | 1276 | -65.103 | -63.154 | -65.105 | -63.137 | 680 |
|  |  | 1278 | -65.103 | -63.150 | -65.108 | -63.143 | 675 |
| Barilari Bay | Outer (BO) | 1300 | -65.782 | -64.863 | -65.767 | -64.850 | 630 |
|  | Inner (BI) | 1295 | -65.940 | -64.640 | -65.943 | -64.654 | 610 |
|  |  | 1297 | -65.943 | -64.622 | -65.948 | -64.631 | 610 |
| Stn B | B-1 | 961 | -64.804 | -65.382 | -64.812 | -65.391 | 606 |
|  |  | 964 | -64.801 | -65.376 | -64.805 | -65.391 | 582 |
|  | B-2 | 1130 | -64.800 | -65.376 | -64.806 | -65.399 | 601 |
|  |  | 1132 | -64.797 | -65.388 | -64.797 | -65.413 | 642 |
|  | B-3 | 1255 | -64.812 | -65.391 | -64.799 | -65.383 | 678 |
|  |  | 1267 | -64.146 | -65.391 | -64.800 | -65.379 | 631 |
| Stn E | E-1 | 976(1) | -65.986 | -67.287 | -65.992 | -67.305 | 590 |
|  |  | 976(2) | -65.986 | -67.287 | -65.992 | -67.305 | 590 |
|  | E-2 | 1091 | -65.976 | -67.300 | -65.980 | -67.282 | 595 |
|  |  | 1103 | -65.921 | -67.472 | -65.932 | -67.434 | 573 |
|  | E-3 | 1217 | -65.977 | -67.294 | -65.983 | -67.274 | 600 |
|  |  | 1219 | -65.981 | -67.301 | -65.975 | -67.285 | 598 |
| Stn F | F-1 | 990 | -66.958 | -69.718 | -66.992 | -69.735 | 584 |
|  |  | 1005 | -66.988 | -69.723 | -66.997 | -69.740 | 610 |
|  | F-2 | 1069 | -66.984 | -69.713 | -66.983 | -69.702 | 586 |
|  |  | 1072 | -66.985 | -69.710 | -66.985 | -69.079 | 581 |
|  | F-3 | 1207 | -66.981 | -69.717 | -66.994 | -69.713 | 578 |
|  |  | 1208 | -66.979 | -69.717 | -66.988 | -69.722 | 579 |
