# Supplementary material for: Megafaunal Communities in Rapidly Warming Fjords along the West Antarctic Peninsula: Hotspots of Abundance and Beta Diversity
Source: PLoS One. 2013 Dec 3;8(12):e77917. doi: 10.1371/journal.pone.0077917 (PMC3848936; doi:10.1371/journal.pone.0077917)
Supplement: Table S3 — Dominant demersal nekton species by percentage of total abundance in WAP fjord basins and at open shelf stations. Data are mean abundances m−2 ± 1 SE using phototransects as replicates. Basins: AI = Andvord Bay inner; AM = Andvord Bay middle; AO = Andvord Bay outer; AMTH = Andvord Bay mouth; FIA = Flandres Bay inner A; FIB = Flandres Bay inner B; FO = Flandres Bay outer; BI = Barilari Bay inner; and BO = Barilari Bay outer. (DOC) [file pone.0077917.s013.doc]

| **FJORD BASINS** | | | | | | | |
| --- | --- | --- | --- | --- | --- | --- | --- |
| **AI** | **Mean (m^-2^)** | **S.E.** | **%** | **FIA** | **Mean (m^-2^)** | **S.E.** | **%** |
| *Ptychogastria polaris* | 1.0 | 0.47 | 43.4 | Mysid sp.1 | 0.2 | 0.06 | 47.4 |
| Krill sp. 1 | 0.6 | 0.09 | 26.0 | *Ptychogastria polaris* | 0.2 | 0.10 | 36.8 |
| Mysid sp. 1 | 0.4 | 0.14 | 19.0 | Medusa sp. 2 | 0.04 | 0.03 | 9.2 |
| Medusa sp. 2 | 0.2 | 0.01 | 7.8 | Krill sp. 2 | 0.03 | 0.01 | 6.6 |
| Chaetognath sp. 1 | 0.01 | 0.01 | 0.5 |  |  |  |  |
| Medusa sp. 3 | 0.01 | 0.01 | 0.3 |  |  |  |  |
| **AM** | **Mean (m^-2^)** | **S.E.** | **%** | **FIB** | **Mean (m^-2^)** | **S.E.** | **%** |
| *Ptychogastria polaris* | 0.6 | 0.01 | 64.4 | Medusa sp. 2 | 0.1 | 0.06 | 37.6 |
| Mysid sp. 1 | 0.3 | 0.04 | 32.5 | Mysid sp. 1 | 0.1 | 0.02 | 36.8 |
| Medusa sp. 2 | 0.02 | 0.02 | 1.9 | Krill sp. 1 | 0.03 | 0.001 | 13.7 |
| Krill sp. 1 | 0.01 | 0.01 | 1.3 | *Ptychogastria polaris* | 0.01 | 0.01 | 4.8 |
|  |  |  |  | Medusa sp. 3 | 0.01 | 0.01 | 4.8 |
|  |  |  |  | Chaetognath sp. 1 | 0.01 | 0.01 | 2.4 |
| **AO** | **Mean (m^-2^)** | **S.E.** | **%** | **FO** | **Mean (m^-2^)** | **S.E.** | **%** |
| Krill sp. 1 | 0.4 | 0.04 | 41.4 | Mysid sp. 1 | 0.1 | 0.01 | 60.7 |
| Mysid sp. 1 | 0.3 | 0.03 | 32.8 | Medusa sp. 2 | 0.04 | 0.04 | 25.0 |
| *Ptychogastria polaris* | 0.3 | 0.04 | 24.7 | Krill sp. 1 | 0.01 | 0.01 | 7.1 |
| Medusa sp. 2 | 0.01 | 0.00 | 1.1 | *Ptychogastria polaris* | 0.01 | 0.00 | 7.1 |
| **AMTH** | **Mean (m^-2^)** | **S.E.** | **%** | **BI** | **Mean (m^-2^)** | **S.E.** | **%** |
| *Ptychogastria polaris* | 0.6 | 0.27 | 44.2 | Krill sp. 1 | 2.0 | 0.48 | 92.2 |
| Krill sp. 1 | 0.5 | 0.40 | 38.0 | Mysid sp. 1 | 0.1 | 0.01 | 6.3 |
| Mysid sp. 1 | 0.2 | 0.01 | 16.1 | Medusa sp. 2 | 0.03 | 0.01 | 1.6 |
| Medusa sp. 2 | 0.02 | 0.01 | 1.7 |  |  |  |  |
|  |  |  |  | **BO** | **Mean (m^-2^)** | **S.E.** | **%** |
|  |  |  |  | Mysid sp. 1 | 0.2 | 0.00 | 61.3 |
|  |  |  |  | Krill sp. 1 | 0.1 | 0.00 | 19.4 |
|  |  |  |  | Medusa sp. 2 | 0.04 | 0.00 | 12.9 |
|  |  |  |  | *Ptychogastria polaris* | 0.02 | 0.00 | 6.5 |
| **OPEN SHELF STATIONS** | | | | | | | |
| **B** | **Mean (m^-2^)** | **S.E.** | **%** | **E** | **Mean (m^-2^)** | **S.E.** | **%** |
| Krill sp. 1 | 0.2 | 0.02 | 93.7 | Krill sp. 1 | 0.2 | 0.05 | 96.3 |
| Mysid sp. 1 | 0.01 | 0.01 | 6.3 | Mysid sp. 1 | 0.01 | 0.00 | 3.7 |
| F | Mean (m^-2^) | S.E. | % |  |  |  |  |
| Krill sp. 1 | 0.2 | 0.03 | 98.4 |  |  |  |  |
| Mysid sp. 1 | 0.004 | 0.06 | 1.6 |  |  |  |  |
