# Supplementary material for: Megafaunal Communities in Rapidly Warming Fjords along the West Antarctic Peninsula: Hotspots of Abundance and Beta Diversity
Source: PLoS One. 2013 Dec 3;8(12):e77917. doi: 10.1371/journal.pone.0077917 (PMC3848936; doi:10.1371/journal.pone.0077917)
Supplement: Table S4 — ANOSIM analysis. ANOSIM pairwise tests between individual fjords and open shelf stations. (DOC) [file pone.0077917.s014.doc]

| **Groups** | **R statistic** | **Significance Level %** | **P-value** | **Possible permutations** | **Actual permutations** | **Number > = Observed** |
| --- | --- | --- | --- | --- | --- | --- |
| Andvord Bay, Flandres Bay | 0.826 | 0.2 | 0.002 | 3003 | 999 | 1 |
| Andvord Bay, Barilari Bay | 1 | 0.6 | 0.006 | 165 | 165 | 1 |
| Andvord Bay, B | 1 | 0.1 | 0.001 | 3003 | 999 | 0 |
| Andvord Bay, E | 1 | 0.1 | 0.001 | 3003 | 999 | 0 |
| Andvord Bay, F | 1 | 0.1 | 0.001 | 3003 | 999 | 0 |
| Flandres Bay, Barilari Bay | 0.623 | 1.2 | 0.012 | 84 | 84 | 1 |
| Flandres Bay, B | 0.998 | 0.2 | 0.002 | 462 | 462 | 1 |
| Flandres Bay, E | 0.994 | 0.2 | 0.002 | 462 | 462 | 1 |
| Flandres Bay, F | 0.998 | 0.2 | 0.002 | 462 | 462 | 1 |
| Barilari Bay, B | 1 | 1.2 | 0.012 | 84 | 84 | 1 |
| Barilari Bay, E | 1 | 1.2 | 0.012 | 84 | 84 | 1 |
| Barilari Bay, F | 1 | 1.2 | 0.012 | 84 | 84 | 1 |
| B, E | 0.913 | 0.2 | 0.002 | 462 | 462 | 1 |
| B, F | 1 | 0.2 | 0.002 | 462 | 462 | 1 |
| E, F | 0.909 | 0.2 | 0.002 | 462 | 462 | 1 |
