# Supplementary material for: Megafaunal Communities in Rapidly Warming Fjords along the West Antarctic Peninsula: Hotspots of Abundance and Beta Diversity
Source: PLoS One. 2013 Dec 3;8(12):e77917. doi: 10.1371/journal.pone.0077917 (PMC3848936; doi:10.1371/journal.pone.0077917)
Supplement: Table S5 — SIMPER analysis of fjords and open shelf stations. Av.Abund: based on 4th root transformed data, Av.Sim = average of the bray curtis similarities between all pairs of sites; Sim/SD = ratio of average contribution (column 2) divided by SD of those contributions across all pairs of samples making up this average - larger number means more consistently contributes to similarity between sites; Contrib% = percentage contribution of total percentage average similarity e.g. 71.20 Andvord Bay; and Cum.% = culminated % contributions in column 4 until cut off % (in this case ∼50%). (DOC) [file pone.0077917.s015.doc]

| **Andvord Bay** | | | | | |
| --- | --- | --- | --- | --- | --- |
| Average similarity = 71.20 | | | | | |
| Species | Av. Abund | Av. Sim | Sim/SD | Contrib% | Cum.% |
| Ampeliscid amphipod sp. 1 | 5.8 | 10.8 | 14.0 | 15.2 | 15.2 |
| *Amythas membranifera* | 5.8 | 10.1 | 4.0 | 14.2 | 29.4 |
| Anemone sp. 2 | 3.8 | 7.2 | 9.4 | 10.2 | 39.6 |
| *Ptychogastria polaris* | 3.6 | 6.4 | 7.9 | 9.0 | 48.5 |
| *Notocrangon antarcticus* | 2.0 | 3.5 | 4.7 | 5.0 | 53.5 |
| **Flandres Bay** | | | | | |
| Average similarity: 50.57 | | | | | |
| Species | Av. Abund | Av. Sim | Sim/SD | Contrib% | Cum.% |
| Pycnogonid sp. 1 | 5.2 | 10.3 | 6.8 | 20.3 | 20.3 |
| *Prionosyllis kerguelensis* | 3.5 | 5.2 | 1.9 | 10.3 | 30.6 |
| Eusirid sp. | 1.7 | 3.4 | 10.4 | 6.8 | 37.4 |
| *Ptychogastria polaris* | 1.8 | 3.3 | 14.1 | 6.6 | 44.0 |
| Zoarcid sp. 1 | 1.2 | 2.4 | 10.3 | 4.8 | 48.8 |
| **Barilari Bay** | | | | | |
| Average similarity: 56.20 | | | | | |
| Species | Av. Abund | Av. Sim | Sim/SD | Contrib% | Cum.% |
| Sabellid sp. 1 | 4.8 | 7.8 | 10.6 | 13.9 | 13.9 |
| Pycnogonid sp. 1  (Small nymphon & Ammothea clausii sp. ) | 3.3 | 6.6 | 6.9 | 11.7 | 25.6 |
| Pycnogonid sp. 5 (large & spindly) | 3.2 | 5.8 | 2.5 | 10.4 | 35.9 |
| Tunicate sp. 5 | 2.8 | 5.1 | 2.7 | 9.0 | 45.0 |
| *Elpidia glacialis* | 2.7 | 4.3 | 1.7 | 7.6 | 52.6 |
| **Station B** | | | | | |
| Average similarity: 63.06 | | | | | |
| Species | Av. Abund | Av. Sim | Sim/SD | Contrib% | Cum.% |
| Pycnogonid sp. 1 | 2.2 | 6.8 | 15.7 | 10.9 | 10.9 |
| Ophiuroid sp. 5 (small, blue central disc) | 1.9 | 6.1 | 13.4 | 9.6 | 20.5 |
| Munnopsid sp. 1 | 1.6 | 5.2 | 11.3 | 8.2 | 28.7 |
| Cerianthid sp. 1 | 1.7 | 5.0 | 3.6 | 8.0 | 36.7 |
| Tunicate sp. 4 | 1.7 | 4.9 | 8.8 | 7.7 | 44.4 |
| Anemone sp. 4 | 1.4 | 4.4 | 9.7 | 6.9 | 51.3 |
| **Station E** | | | | | |
| Average similarity = 49.17 | | | | | |
| Species | Av. Abund | Av. Sim | Sim/SD | Contrib% | Cum.% |
| Ampeliscid amphipod sp. 1 | 2.4 | 8.7 | 3.7 | 17.7 | 17.7 |
| *Protelpidia murrayi* | 2.0 | 7.6 | 5.7 | 15.4 | 33.1 |
| Anemone sp. 10 (*Bolocera kerguelensis*?) | 1.3 | 4.2 | 6.0 | 8.5 | 41.6 |
| Scale worm sp. 2 (Blue polynoid) | 1.2 | 4.1 | 4.3 | 8.4 | 50.0 |
| *Elpidia glacialis* | 1.5 | 3.4 | 1.3 | 6.9 | 56.9 |
| **Station F** | | | | | |
| Average similarity = 63.00 | | | | | |
| Species | Av. Abund | Av. Sim | Sim/SD | Contrib% | Cum.% |
| Ampeliscid amphipod sp. 1 | 2.7 | 9.9 | 13.8 | 15.7 | 15.7 |
| *Protelpidia murrayi* | 2.6 | 9.9 | 8.9 | 15.6 | 31.3 |
| *Rhipidothuria racovitzai* | 2.8 | 8.9 | 3.7 | 14.1 | 45.4 |
| Pycnogonid sp. 5 (large & spindly) | 1.5 | 5.6 | 9.7 | 9.0 | 54.3 |
| Munnopsid sp. 1 | 1.6 | 5.6 | 5.8 | 8.9 | 63.2 |
