# Supplementary material for: Megafaunal Communities in Rapidly Warming Fjords along the West Antarctic Peninsula: Hotspots of Abundance and Beta Diversity
Source: PLoS One. 2013 Dec 3;8(12):e77917. doi: 10.1371/journal.pone.0077917 (PMC3848936; doi:10.1371/journal.pone.0077917)
Supplement: Table S6 — SIMPER analysis of Andvord Bay versus open shelf stations. Av.Abund = based on 4th root transformed data; Av.Diss = average of the bray curtis dissimilarities between all pairs of sites; Diss/SD = ratio of average contribution (column 2) divided by SD of those contributions across all pairs of samples making up this average - larger number means more consistently contributes to dissimilarity between sites; Contrib% = percentage contribution of total percentage average dissimilarity e.g. 88.0 Andvord Bay & B; and Cum.% = culminated % contributions in column 5 until cut off % (in this case ∼50%). (DOC) [file pone.0077917.s016.doc]

| **Andvord Bay and Stn B** | | | | | | |
| --- | --- | --- | --- | --- | --- | --- |
| Average dissimilarity = 88.0 | | | | | | |
|  | Andvord Bay | Stn B |  |  |  |  |
| Species | Av.Abund | Av.Abund | Av.Diss | Diss/SD | Contrib% | Cum% |
| *Amythas membranifera* | 5.8 | 0.0 | 7.4 | 4.1 | 8.3 | 8.3 |
| Ampeliscid amphipod sp. 1 | 5.8 | 0.0 | 7.3 | 10.9 | 8.3 | 16.6 |
| *Ptychogastria polaris* | 3.6 | 0.0 | 4.5 | 6.6 | 5.1 | 21.7 |
| Anemone sp. 2 | 3.8 | 0.8 | 3.9 | 3.8 | 4.4 | 26.1 |
| Pycnogonid sp. 1 | 0.0 | 2.2 | 2.7 | 10.9 | 3.1 | 29.2 |
| *Notocrangon antarcticus* | 2.0 | 0.0 | 2.5 | 4.4 | 2.9 | 32.1 |
| Ophiuroid sp. 5 (small, blue central disc) | 0.0 | 1.9 | 2.4 | 7.9 | 2.8 | 34.9 |
| Terebellid sp. 1 | 1.7 | 0.0 | 2.2 | 3.6 | 2.4 | 37.3 |
| Tunicate sp. 4 | 0.0 | 1.7 | 2.1 | 6.6 | 2.3 | 39.7 |
| Munnopsid sp. 1 | 0.0 | 1.6 | 2.0 | 12.7 | 2.3 | 41.9 |
| Anemone sp. 5 | 1.6 | 0.0 | 2.0 | 6.5 | 2.3 | 44.2 |
| *Peniagone vignioni* | 0.0 | 1.5 | 1.8 | 6.2 | 2.1 | 46.2 |
| Hydroid sp. 1 | 1.4 | 0.0 | 1.8 | 2.2 | 2.0 | 48.2 |
| *Limopsis marionensis* | 0.0 | 1.3 | 1.7 | 3.8 | 1.9 | 50.2 |
| **Andvord Bay and Stn E** | | | | | | |
| Average dissimilarity = 84.2 | | | | | | |
|  | Andvord Bay | Stn E |  |  |  |  |
| Species | Av.Abund | Av.Abund | Av.Diss | Diss/SD | Contrib% | Cum% |
| *Amythas membranifera* | 5.8 | 0.0 | 7.7 | 3.8 | 9.2 | 9.2 |
| *Ptychogastria polaris* | 3.6 | 0.0 | 4.7 | 5.9 | 5.6 | 14.8 |
| Ampeliscid amphipod sp. 1 | 5.8 | 2.4 | 4.5 | 6.7 | 5.4 | 20.2 |
| Anemone sp. 2 | 3.8 | 0.4 | 4.4 | 4.3 | 5.3 | 25.5 |
| *Protelpidia murrayi* | 0.0 | 2.0 | 2.7 | 9.1 | 3.2 | 28.6 |
| *Notocrangon antarcticus* | 2.0 | 0.0 | 2.7 | 4.1 | 3.2 | 31.8 |
| Terebellid sp. 1 | 1.7 | 0.0 | 2.3 | 3.5 | 2.7 | 34.5 |
| Anemone sp. 5 | 1.6 | 0.0 | 2.1 | 5.8 | 2.5 | 37.0 |
| Zoarcid sp. 1 | 1.6 | 0.2 | 1.9 | 2.7 | 2.3 | 39.3 |
| *Elpidia glacialis* | 0.0 | 1.5 | 1.9 | 1.9 | 2.2 | 41.5 |
| Hydroid sp. 1 | 1.4 | 0.0 | 1.9 | 2.1 | 2.2 | 43.7 |
| Anemone sp. 10 (*Bolocera kerguelensis*?) | 0.0 | 1.3 | 1.6 | 5.8 | 1.9 | 45.6 |
| Scale worm sp. 2 (Blue polynoid) | 0.0 | 1.2 | 1.6 | 4.0 | 1.9 | 47.6 |
| Eusirid sp. | 1.5 | 0.3 | 1.6 | 2.2 | 1.9 | 49.4 |
| *Ophionotus victoriae* | 1.1 | 0.0 | 1.6 | 0.9 | 1.8 | 51.3 |
| **Andvord Bay and Stn F** | | | | | | |
| Average dissimilarity = 84.3 | | | | | | |
|  | Andvord Bay | Stn F |  |  |  |  |
| Species | Av.Abund | Av.Abund | Av.Diss | Diss/SD | Contrib% | Cum% |
| *Amythas membranifera* | 5.8 | 0.0 | 7.7 | 4.1 | 9.2 | 9.2 |
| *Ptychogastria polaris* | 3.6 | 0.0 | 4.7 | 7.0 | 5.6 | 14.8 |
| Ampeliscid amphipod sp. 1 | 5.8 | 2.7 | 4.1 | 5.9 | 4.9 | 19.7 |
| Anemone sp. 2 | 3.8 | 0.7 | 4.0 | 4.8 | 4.8 | 24.5 |
| *Rhipidothuria racovitzai* | 0.0 | 2.8 | 3.7 | 3.5 | 4.4 | 28.8 |
| *Protelpidia murrayi* | 0.0 | 2.6 | 3.5 | 9.7 | 4.1 | 33.0 |
| *Notocrangon antarcticus* | 2.0 | 0.0 | 2.7 | 4.4 | 3.2 | 36.1 |
| Terebellid sp. 1 | 1.7 | 0.0 | 2.3 | 3.7 | 2.7 | 38.8 |
| *Peniagone vignioni* | 0.0 | 1.6 | 2.1 | 5.0 | 2.5 | 41.3 |
| Anemone sp. 5 | 1.6 | 0.0 | 2.1 | 6.8 | 2.5 | 43.8 |
| Munnopsid sp. 1 | 0.0 | 1.6 | 2.0 | 6.8 | 2.4 | 46.2 |
| Pycnogonid sp. 5 (large & spindly) | 0.0 | 1.5 | 2.0 | 7.8 | 2.4 | 48.6 |
| Munnopsid sp. 2 | 0.0 | 1.5 | 1.9 | 1.9 | 2.2 | 50.8 |
