# Supplementary material for: Megafaunal Communities in Rapidly Warming Fjords along the West Antarctic Peninsula: Hotspots of Abundance and Beta Diversity
Source: PLoS One. 2013 Dec 3;8(12):e77917. doi: 10.1371/journal.pone.0077917 (PMC3848936; doi:10.1371/journal.pone.0077917)
Supplement: Table S7 — SIMPER analysis of Flandres Bay versus open shelf stations. Av.Abund = based on 4th root transformed data; Av.Diss = average of the bray curtis dissimilarities between all pairs of sites; Diss/SD = ratio of average contribution (column 2) divided by SD of those contributions across all pairs of samples making up this average - larger number means more consistently contributes to dissimilarity between sites; Contrib% = percentage contribution of total percentage average dissimilarity e.g. 81.9 Flandres Bay & B; and Cum.% = culminated % contributions in column 5 until cut of % (in this case ∼50%). (DOC) [file pone.0077917.s017.doc]

| **Flandres Bay and Stn B** |  |  |  |  |  |  |
| --- | --- | --- | --- | --- | --- | --- |
| Average dissimilarity = 81.9 |  |  |  |  |  |  |
|  | Flandres Bay | Stn B |  |  |  |  |
| Species | Av.Abund | Av.Abund | Av.Diss | Diss/SD | Contrib% | Cum% |
| *Prionosyllis kerguelensis* | 3.5 | 0.2 | 4.2 | 2.1 | 5.1 | 5.1 |
| Pycnogonid sp. 1 | 5.2 | 2.2 | 4.0 | 3.7 | 4.8 | 10.0 |
| Ophiuroid sp. 5 (small, blue central disc) | 0.0 | 1.9 | 2.5 | 8.2 | 3.1 | 13.0  15.8 |
| *Ptychogastria polaris* | 1.8 | 0.0 | 2.3 | 8.0 | 2.8 | 15.8 |
| Cerianthid sp. 1 | 0.0 | 1.7 | 2.2 | 4.2 | 2.7 | 18.5 |
| Tunicate sp. 4 | 0.0 | 1.7 | 2.1 | 6.9 | 2.6 | 21.1 |
| Anemone sp. 5 | 1.6 | 0.0 | 2.1 | 1.3 | 2.5 | 23.6 |
| Munnopsid sp. 1 | 0.0 | 1.6 | 2.1 | 14.3 | 2.5 | 26.2 |
| *Ophionotus victoriae* | 1.4 | 0.2 | 1.9 | 0.8 | 2.3 | 28.5 |
| *Peniagone vignioni* | 0.0 | 1.5 | 1.9 | 6.4 | 2.3 | 30.8 |
| *Limopsis marionensis* | 0.0 | 1.3 | 1.8 | 3.9 | 2.1 | 32.9 |
| Eusirid sp. | 1.7 | 0.4 | 1.7 | 2.1 | 2.1 | 35.0 |
| Tunicate sp. 8 (*Synoicum* sp.?) | 0.0 | 1.3 | 1.7 | 1.3 | 2.0 | 37.0 |
| Anemone sp. 9 | 1.3 | 0.0 | 1.6 | 1.3 | 2.0 | 39.0 |
| Demospongiae sp. 1 | 1.1 | 0.0 | 1.5 | 1.2 | 1.9 | 40.8 |
| Anemone sp. 4 | 1.6 | 1.4 | 1.4 | 1.8 | 1.8 | 42.6 |
| Asteroid sp. 2 (small & white) | 1.1 | 0.3 | 1.4 | 1.1 | 1.7 | 44.3 |
| *Cuenotaster involutus* | 1.1 | 0.0 | 1.4 | 2.1 | 1.7 | 46.0 |
| Bonellid echiuran sp. 1 | 1.0 | 0.0 | 1.4 | 2.0 | 1.7 | 47.7 |
| Anemone sp. 2 | 1.3 | 0.8 | 1.4 | 1.5 | 1.7 | 49.3 |
| *Harpovoluta charcoti* | 1.1 | 0.0 | 1.4 | 1.3 | 1.7 | 51.0 |
| **Flandres Bay and Stn E** |  |  |  |  |  |  |
| Average dissimilarity = 84.5 |  |  |  |  |  |  |
|  | Flandres Bay | Stn E |  |  |  |  |
| Species | Av.Abund | Av.Abund | Av.Diss | Diss/SD | Contrib% | Cum% |
| Pycnogonid sp. 1 | 5.2 | 1.2 | 5.6 | 3.2 | 6.6 | 6.6 |
| *Prionosyllis kerguelensis* | 3.5 | 0.2 | 4.5 | 2.1 | 5.3 | 11.9 |
| *Protelpidia murrayi* | 0.0 | 2.0 | 2.8 | 9.5 | 3.3 | 15.2 |
| Ampeliscid amphipod sp. 1 | 0.4 | 2.4 | 2.7 | 2.8 | 3.2 | 18.3 |
| *Ptychogastria polaris* | 1.8 | 0.0 | 2.4 | 6.7 | 2.8 | 21.2 |
| Anemone sp. 5 | 1.6 | 0.0 | 2.2 | 1.3 | 2.6 | 23.7 |
| *Elpidia glacialis* | 0.0 | 1.5 | 2.0 | 1.9 | 2.3 | 26.1 |
| *Ophionotus victoriae* | 1.4 | 0.0 | 2.0 | 0.7 | 2.3 | 28.4 |
| Anemone sp. 4 | 1.6 | 0.4 | 1.9 | 1.3 | 2.3 | 30.7 |
| Eusirid sp. | 1.7 | 0.3 | 1.9 | 2.4 | 2.3 | 32.9 |
| Anemone sp. 9 | 1.3 | 0.0 | 1.7 | 1.3 | 2.0 | 34.9 |
| Demospongiae sp. 1 | 1.1 | 0.0 | 1.6 | 1.2 | 1.9 | 36.8 |
| Anemone sp. 2 | 1.3 | 0.4 | 1.6 | 1.4 | 1.8 | 38.7 |
| Anemone sp. 10 (*Bolocera kerguelensis*?) | 0.2 | 1.3 | 1.5 | 2.5 | 1.8 | 40.4 |
| Asteroid sp. 2 (small & white) | 1.1 | 0.0 | 1.5 | 0.9 | 1.8 | 42.2 |
| Zoarcid sp. 1 | 1.2 | 0.2 | 1.5 | 2.5 | 1.7 | 43.9 |
| *Cuenotaster involutus* | 1.1 | 0.0 | 1.5 | 2.1 | 1.7 | 45.6 |
| *Harpovoluta charcoti* | 1.1 | 0.0 | 1.4 | 1.3 | 1.7 | 47.3 |
| *Isosycionis alba* | 1.1 | 0.0 | 1.4 | 1.3 | 1.7 | 49.0 |
| Asteroid sp. 3 (*Diplasterias brucei*?) | 1.0 | 0.0 | 1.4 | 0.9 | 1.7 | 50.7 |
| **Flandres Bay and Stn F** |  |  |  |  |  |  |
| Average dissimilarity = 82.8 |  |  |  |  |  |  |
|  | Flandres Bay | Stn F |  |  |  |  |
| Species | Av.Abund | Av.Abund | Av.Diss | Diss/SD | Contrib% | Cum% |
| Pycnogonid sp. 1 | 5.2 | 0.6 | 6.3 | 4.3 | 7.6 | 7.6 |
| *Prionosyllis kerguelensis* | 3.5 | 0.0 | 4.7 | 2.4 | 5.6 | 13.3 |
| *Protelpidia murrayi* | 0.0 | 2.6 | 3.6 | 10.3 | 4.4 | 17.6 |
| *Rhipidothuria racovitzai* | 1.1 | 2.8 | 3.4 | 2.6 | 4.1 | 21.7 |
| Ampeliscid amphipod sp. 1 | 0.4 | 2.7 | 3.1 | 3.9 | 3.7 | 25.4 |
| *Ptychogastria polaris* | 1.8 | 0.0 | 2.4 | 8.8 | 2.9 | 28.3 |
| *Peniagone vignioni* | 0.0 | 1.6 | 2.2 | 5.0 | 2.7 | 31.0 |
| Anemone sp. 5 | 1.6 | 0.0 | 2.2 | 1.3 | 2.6 | 33.6 |
| Munnopsid sp. 1 | 0.0 | 1.6 | 2.1 | 7.0 | 2.6 | 36.2 |
| *Ophionotus victoriae* | 1.4 | 0.4 | 2.1 | 0.9 | 2.5 | 38.7 |
| Munnopsid sp. 2 | 0.0 | 1.5 | 2.0 | 1.9 | 2.4 | 41.1 |
| Anemone sp. 4 | 1.6 | 0.5 | 1.9 | 1.5 | 2.3 | 43.3 |
| Eusirid sp. | 1.7 | 0.4 | 1.8 | 2.3 | 2.2 | 45.6 |
| Anemone sp. 9 | 1.3 | 0.0 | 1.7 | 1.3 | 2.1 | 47.6 |
| Demospongiae sp. 1 | 1.1 | 0.0 | 1.6 | 1.2 | 1.9 | 49.5 |
| Asteroid sp. 2 (small & white) | 1.1 | 0.0 | 1.5 | 0.9 | 1.8 | 51.3 |
