# Supplementary material for: Megafaunal Communities in Rapidly Warming Fjords along the West Antarctic Peninsula: Hotspots of Abundance and Beta Diversity
Source: PLoS One. 2013 Dec 3;8(12):e77917. doi: 10.1371/journal.pone.0077917 (PMC3848936; doi:10.1371/journal.pone.0077917)
Supplement: Table S8 — SIMPER analysis of Barilari Bay versus open shelf stations. Av.Abund = based on 4th root transformed data; Av.Diss = average of the bray curtis dissimilarities between all pairs of sites; Diss/SD = ratio of average contribution (column 2) divided by SD of those contributions across all pairs of samples making up this average - larger number means more consistently contributes to dissimilarity between sites; Contrib% = percentage contribution of total percentage average dissimilarity e.g. 82.3 Barilari & B; and Cum.% = culminated % contributions in column 5 until cut of % (in this case ∼50%). (DOC) [file pone.0077917.s018.doc]

| **Barilari Bay and Stn B** |  |  |  |  |  |  |
| --- | --- | --- | --- | --- | --- | --- |
| Average dissimilarity = 82.3 |  |  |  |  |  |  |
|  | Barilari Bay | Stn B |  |  |  |  |
| Species | Av.Abund | Av.Abund | Av.Diss | Diss/SD | Contrib% | Cum% |
| Sabellid sp. 1 | 4.8 | 1.0 | 4.9 | 3.1 | 5.9 | 5.9 |
| Pycnogonid sp. 5 (large & spindly) | 3.2 | 0.2 | 4.1 | 2.9 | 5.0 | 10.9 |
| *Elpidia glacialis* | 2.7 | 0.0 | 3.7 | 2.4 | 4.5 | 15.4 |
| Tunicate sp. 5 | 2.8 | 0.0 | 3.7 | 3.6 | 4.5 | 19.8 |
| *Prionosyllis kerguelensis* | 2.7 | 0.2 | 3.1 | 1.9 | 3.8 | 23.6 |
| Tunicate sp. 3 | 1.9 | 0.0 | 2.6 | 4.6 | 3.1 | 26.7 |
| Ophiuroid sp. 5 (small, blue central disc) | 0.0 | 1.9 | 2.5 | 6.9 | 3.1 | 29.8 |
| Cerianthid sp. 1 | 0.0 | 1.7 | 2.2 | 3.9 | 2.7 | 32.5 |
| Tunicate sp. 4 | 0.0 | 1.7 | 2.1 | 6.1 | 2.6 | 35.1 |
| Munnopsid sp. 1 | 0.0 | 1.6 | 2.1 | 9.6 | 2.5 | 37.6 |
| *Rhipidothuria racovitzai* | 1.5 | 0.0 | 2.0 | 3.2 | 2.4 | 40.0 |
| Anemone sp. 4 | 0.0 | 1.4 | 1.9 | 6.3 | 2.3 | 42.3 |
| *Limopsis marionensis* | 0.0 | 1.3 | 1.8 | 3.6 | 2.1 | 44.5 |
| Irregular urchin sp. 1 | 1.4 | 0.0 | 1.7 | 1.3 | 2.0 | 46.5 |
| *Marseniopsis* sp. 1 | 0.0 | 1.2 | 1.6 | 5.8 | 1.9 | 48.4 |
| *Psilaster charcoti* | 1.2 | 0.0 | 1.6 | 8.6 | 1.9 | 50.3 |
| **Barilari Bay and Stn E** |  |  |  |  |  |  |
| Average dissimilarity = 78.1 |  |  |  |  |  |  |
|  | Barilari Bay | Stn E |  |  |  |  |
| Species | Av.Abund | Av.Abund | Av.Diss | Diss/SD | Contrib% | Cum% |
| Sabellid sp. 1 | 4.8 | 0.6 | 5.8 | 2.9 | 7.4 | 7.4 |
| Pycnogonid sp. 5 (large & spindly) | 3.2 | 0.3 | 4.1 | 2.5 | 5.3 | 12.7 |
| Tunicate sp. 5 | 2.8 | 0.0 | 3.9 | 3.3 | 5.0 | 17.6 |
| *Prionosyllis kerguelensis* | 2.7 | 0.2 | 3.3 | 2.0 | 4.3 | 21.9 |
| Ampeliscid amphipod sp. 1 | 0.0 | 2.4 | 3.3 | 4.5 | 4.2 | 26.1 |
| Pycnogonid sp. 1 | 3.3 | 1.2 | 2.9 | 2.3 | 3.8 | 29.8 |
| *Elpidia glacialis* | 2.7 | 1.5 | 2.2 | 1.3 | 2.8 | 32.6 |
| *Rhipidothuria racovitzai* | 1.5 | 0.0 | 2.1 | 3.1 | 2.7 | 35.3 |
| Tunicate sp. 3 | 1.9 | 0.6 | 2.0 | 2.0 | 2.5 | 37.8 |
| *Pareledone charcoti* | 1.3 | 0.0 | 1.7 | 8.8 | 2.2 | 40.0 |
| Anemone sp. 10 (*Bolocera kerguelensis*?) | 0.0 | 1.3 | 1.7 | 5.3 | 2.2 | 42.2 |
| *Psilaster charcoti* | 1.2 | 0.0 | 1.6 | 6.7 | 2.1 | 44.3 |
| Irregular urchin sp. 1 | 1.4 | 0.3 | 1.6 | 1.4 | 2.1 | 46.4 |
| Enteropneust sp. 1 | 1.0 | 0.2 | 1.4 | 1.3 | 1.9 | 48.2 |
| *Cuenotaster involutus* | 1.0 | 0.0 | 1.4 | 6.7 | 1.8 | 50.0 |
| Tunicate sp. 8 (*Synoicum* sp.?) | 0.6 | 0.9 | 1.3 | 1.3 | 1.7 | 51.6 |
| **Barilari Bay and Stn F** |  |  |  |  |  |  |
| Average dissimilarity = 77.9 |  |  |  |  |  |  |
|  | Barilari Bay | Stn F |  |  |  |  |
| Species | Av.Abund | Av.Abund | Av.Diss | Diss/SD | Contrib% | Cum% |
| Sabellid sp. 1 | 4.8 | 0.0 | 6.5 | 4.4 | 8.3 | 8.3 |
| *Elpidia glacialis* | 2.7 | 0.0 | 3.9 | 2.4 | 5.0 | 13.1 |
| Tunicate sp. 5 | 2.8 | 0.0 | 3.9 | 3.6 | 5.0 | 18.2 |
| Ampeliscid amphipod sp. 1 | 0.0 | 2.7 | 3.7 | 8.7 | 4.7 | 22.9 |
| Pycnogonid sp. 1 | 3.3 | 0.6 | 3.7 | 3.2 | 4.7 | 27.6 |
| *Prionosyllis kerguelensis* | 2.7 | 0.0 | 3.5 | 2.4 | 4.5 | 32.3 |
| Tunicate sp. 3 | 1.9 | 0.0 | 2.7 | 4.6 | 3.5 | 35.6 |
| Pycnogonid sp. 5 (large & spindly) | 3.2 | 1.5 | 2.4 | 2.0 | 3.1 | 38.8 |
| Munnopsid sp. 1 | 0.0 | 1.6 | 2.1 | 6.0 | 2.8 | 41.5 |
| Munnopsid sp. 2 | 0.0 | 1.5 | 2.0 | 1.8 | 2.5 | 44.0 |
| *Protelpidia murrayi* | 1.2 | 2.6 | 1.9 | 2.0 | 2.5 | 46.5 |
| *Peniagone vignioni* | 0.3 | 1.6 | 1.8 | 2.0 | 2.3 | 48.8 |
| *Rhipidothuria racovitzai* | 1.5 | 2.8 | 1.8 | 1.6 | 2.3 | 51.2 |
