# Supplementary material for: Megafaunal Communities in Rapidly Warming Fjords along the West Antarctic Peninsula: Hotspots of Abundance and Beta Diversity
Source: PLoS One. 2013 Dec 3;8(12):e77917. doi: 10.1371/journal.pone.0077917 (PMC3848936; doi:10.1371/journal.pone.0077917)
Supplement: Table S9 — Shared and unique species inventory. List of shared and unique species combining all WAP fjords and open shelf stations observed during this study. *Stylocordyla chupachups (Porifera: Hadromerida) previously reported as S. borealis (Lovén, 1868) in Uriz M-J, Gili J-M, Orejas C, Perez-Porro A-R (2011) Do bipolar distributions exist in marine sponges? Stylocordyla chupachups sp. nv. (Porifera: Hadromerida) from the Weddell Sea (Antarctic), previously reported as S. borealis (Lovén, 1868). Polar Biology 34: 243–255. (DOC) [file pone.0077917.s019.doc]

| **Shared Species** | | **Fjord Exclusive Species** | |
| --- | --- | --- | --- |
| Fishes | Invertebrates | Fishes | Invertebrates |
| *Chaenocephalus aceratus* | *Ophiosparte gigas* | Antarctic silverfish (*Pleuragramma antarcticum*?) | Gastropod sp. 1 |
| Fish sp. 1 | Ophiuroid sp. 2 | *Channichthyidae* sp. | Hexactinellid sp. 3 (*Rosella nuda*?) |
| *Notothenia corriceps* | *Pareledone charcoti* | Fish sp. 9 | Hydroid sp. 1 |
| Zoarcid sp. 1 | *Peniagone vignioni* | Invertebrates | *Notocrangon antarcticus* |
| Invertebrates | *Phyllochaetopterus* sp. | *Aforia magnifica* | *Parborlasia corrugatus* |
| Ampeliscid amphipod sp. 1 | *Prionosyllis kerguelensis* | *Amythas membranifera* | *Porania antarctica* |
| Anemone sp. 2 | *Promachocrinus kerguelensis* | Anemone sp. 5 | Primnoid octocoral sp. 1 |
| Anemone sp. 4 | *Protelpidia murrayi* | Anemone sp. 7 | *Psilaster charcoti* |
| Anemone sp. 6 | Pycnogonid sp. 1 | Anemone sp. 8 | *Ptychogastria polaris* |
| Anemone sp. 10 (*Bolocera kerguelensis*?) | Pycnogonid sp. 3 (*Colossendeis* sp.) | Anemone sp. 9 | Pycnogonid sp. 2 (*Pentanymphon*) |
| Asteroid sp. 1 | Pycnogonid sp. 5 (large & spindly) | Asteroid sp. 6 (*Perknaster aurorae*) | *Pyura bouvetensis* |
| Asteroid sp. 2 (small & white) | *Rhipidothuria racovitzai* | *Bathyplotes bongraini* | Scale worm sp. 1 (Brown polynoid) |
| Asteroid sp. 3 (*Diplasterias brucei*?) | Sabellid sp. 1 | Bivalve sp. 1 | Sea pen sp. 1 |
| Bonellid echiuran sp. 1 | Scale worm sp. 2 (Blue polynoid) | Bivalve sp. 2 | Sea pen sp. 2 |
| Cerianthid sp. 1 | *Sterechinus* sp. | Crinoid sp. 2 | Terebellid sp. 1 |
| *Ctenocidaris perrieri* | *Stylocordyla chupachups** | *Cuenotaster involutus* | Tunicate sp. 1 |
| Cup coral sp. 1 | Tunicate sp. 3 | Demospongiae sp. 1 | Tunicate sp. 2 (*Cnemidocarpa* sp.?) |
| *Elpidia glacialis* | Tunicate sp. 8 (*Synoicum* sp.?) | Demospongiae sp. 2 | Tunicate sp. 5 |
| Enteropneust sp. 1 | *Umbellula* sp. 1 | Demospongiae sp. 5 (*Haliclona* sp.?) | Tunicate sp. 6 (*Molgula pedunculata*?) |
| Eusirid sp. |  | Demospongiae sp. 6 (*Kirkpatrickia variolosa*?) |  |
| Flabelligerid sp. 1 |  | **Shelf Exclusive Species** | |
| *Fungiacyanthus* sp. |  | Fishes | Invertebrates |
| *Harpovoluta charcoti* |  | Artedraconidae sp. 1 (*Histodraco velifer*?) | Cirroteuthid sp. |
| Hexactinellid sp. 1 |  | Fish sp. 8 | Crinoid sp. 3 |
| Hexactinellid sp. 4 (*Rosella racovitzae*?) |  | Fish sp. 11 | Ctenophore, benthic sp. 1 (*Lyrocteis flavopallidus*?) |
| Holothurian sp. 1 (feeding tentacles present) |  | Zoarcid sp. 2 | *Flabellum impensum* |
| Hormathid anemone sp. |  | Invertebrates | Irregular urchin sp. 3 |
| Hydroid sp. 2 |  | Anemone sp. 11 | Munnopsid sp. 1 |
| Irregular urchin sp. 1 |  | Anemone sp. 12 | Munnopsid sp. 2 |
| Irregular urchin sp. 2 |  | *Anthomastus bathyproctus* | *Limopsis marionensis* |
| Irregular urchin sp. 4 |  | *Anthomastus* sp. 2 | Ophiuroid sp. 5 (small, blue central disc) |
| *Isosycionis alba* |  | Aphroditid sp. 1 | Sea pen sp. 3 |
| *Marseniopsis* sp. 1 |  | Asteroid sp. 8 | Tunicate sp. 4 |
| Nemertean sp. 1 |  | *Ceratoserolis meridionalis* | Tunicate sp. 7 |
| *Ophionotus victoriae* |  | *Cinachyra antarctica* |  |
