# Supplementary material for: Megafaunal Communities in Rapidly Warming Fjords along the West Antarctic Peninsula: Hotspots of Abundance and Beta Diversity
Source: PLoS One. 2013 Dec 3;8(12):e77917. doi: 10.1371/journal.pone.0077917 (PMC3848936; doi:10.1371/journal.pone.0077917)
Supplement: Table S10 — Differences in estimated total epibenthic megafaunal species richness between fjords and open shelf stations. Higher values in the fjords are indicated by a “+”; lower values in the fjords by a “−”. Differences between fjords and the open shelf as a whole (Stations B, E and F) were tested using the Kruskal-Wallis test. Pairwise comparisons between individual fjords and individual open shelf stations were not addressed as no significant difference was identified between fjords versus the open shelf as a whole. ****P<0.0001, ***P<0.001, **P<0.01, *P<0.05 and N.S. (Non-significant) P>0.05. (DOC) [file pone.0077917.s020.doc]

|  | **Estimated total species richness** | | | | |
| --- | --- | --- | --- | --- | --- |
|  | **Bootstrap** | | |  | **Jackknife 2** |
| **Andvord Bay** |  |  |  | |  |
| Fjord vs open shelf stations B, E & F | + | N.S. | + | | N.S. |
| **Flandres Bay** |  |  |  | |  |
| Fjord vs open shelf stations B, E & F | + | N.S. | + | | N.S. |
| **Barilari Bay** |  |  |  | |  |
| Fjord vs open shelf stations B, E & F | + | N.S. | + | | N.S. |
